# Investigating causal relations between sleep traits and risk of breast cancer in women: mendelian randomisation study

**DOI:** 10.1136/bmj.l2327

**Published:** 2019-06-26

**Authors:** Rebecca C Richmond, Emma L Anderson, Hassan S Dashti, Samuel E Jones, Jacqueline M Lane, Linn Beate Strand, Ben Brumpton, Martin K Rutter, Andrew R Wood, Kurt Straif, Caroline L Relton, Marcus Munafò, Timothy M Frayling, Richard M Martin, Richa Saxena, Michael N Weedon, Debbie A Lawlor, George Davey Smith

**Affiliations:** 1 MRC Integrative Epidemiology Unit at the University of Bristol, Bristol, UK; 2 Population Health Sciences, Bristol Medical School, University of Bristol, Bristol, UK; 3 Centre for Genomic Medicine, Massachusetts General Hospital, Harvard Medical School, Boston, MA, USA; 4 Program in Medical and Population Genetics, Broad Institute, Cambridge, MA, USA; 5 Genetics of Complex Traits, University of Exeter Medical School, Exeter, UK; 6 K.G. Jebsen Centre for Genetic Epidemiology, Department of Public Health and Nursing, Faculty of Medicine and Health sciences, Norwegian University of Science and Technology, NTNU, Trondheim, Norway; 7 Clinic of Thoracic and Occupational Medicine, St Olav’s Hospital, Trondheim University Hospital, Trondheim, Norway; 8 Division of Endocrinology, Diabetes and Gastroenterology, School of Medical Sciences, Faculty of Biology, Medicine and Health, University of Manchester, Manchester, UK; 9 Manchester Diabetes Centre, Manchester University NHS Foundation Trust, Manchester Academic Health Science Centre, Manchester, Manchester, UK; 10 International Agency for Research on Cancer, Lyon, France; 11 School of Experimental Psychology, University of Bristol, Bristol, UK; 12 National Institute for Health Research (NIHR) Bristol Biomedical Research Centre, University Hospitals Bristol NHS Foundation Trust and the University of Bristol, Bristol, UK; 13 Department of Anaesthesia, Critical Care and Pain Medicine, Massachusetts General Hospital, Boston, MA, USA; 14 Division of Sleep and Circadian Disorders, Brigham and Women’s Hospital, Harvard Medical School, Boston, MA, USA

## Abstract

**Objective:**

To examine whether sleep traits have a causal effect on risk of breast cancer.

**Design:**

Mendelian randomisation study.

**Setting:**

UK Biobank prospective cohort study and Breast Cancer Association Consortium (BCAC) case-control genome-wide association study.

**Participants:**

156 848 women in the multivariable regression and one sample mendelian randomisation (MR) analysis in UK Biobank (7784 with a breast cancer diagnosis) and 122 977 breast cancer cases and 105 974 controls from BCAC in the two sample MR analysis.

**Exposures:**

Self reported chronotype (morning or evening preference), insomnia symptoms, and sleep duration in multivariable regression, and genetic variants robustly associated with these sleep traits.

**Main outcome measure:**

Breast cancer diagnosis.

**Results:**

In multivariable regression analysis using UK Biobank data on breast cancer incidence, morning preference was inversely associated with breast cancer (hazard ratio 0.95, 95% confidence interval 0.93 to 0.98 per category increase), whereas there was little evidence for an association between sleep duration and insomnia symptoms. Using 341 single nucleotide polymorphisms (SNPs) associated with chronotype, 91 SNPs associated with sleep duration, and 57 SNPs associated with insomnia symptoms, one sample MR analysis in UK Biobank provided some supportive evidence for a protective effect of morning preference on breast cancer risk (0.85, 0.70, 1.03 per category increase) but imprecise estimates for sleep duration and insomnia symptoms. Two sample MR using data from BCAC supported findings for a protective effect of morning preference (inverse variance weighted odds ratio 0.88, 95% confidence interval 0.82 to 0.93 per category increase) and adverse effect of increased sleep duration (1.19, 1.02 to 1.39 per hour increase) on breast cancer risk (both oestrogen receptor positive and oestrogen receptor negative), whereas evidence for insomnia symptoms was inconsistent. Results were largely robust to sensitivity analyses accounting for horizontal pleiotropy.

**Conclusions:**

Findings showed consistent evidence for a protective effect of morning preference and suggestive evidence for an adverse effect of increased sleep duration on breast cancer risk.

## Introduction

In 2007 the World Health Organization’s International Agency for Research on Cancer classified shift work that involves circadian disruption as being probably carcinogenic to humans.^1^ Disturbed sleep, exposure to light at night, and exposure to other lifestyle factors have been proposed as possible underlying mechanisms.^2^ ^3^ ^4^ Although much of the literature on breast cancer risk has focused on the potentially adverse effects of night shift work and exposure to light at night, less investigation has been done into the potential adverse effects of sleep disruption and traits such as chronotype (morning or evening preference), sleep duration, and insomnia.^5^

In a meta-analysis of 28 studies, strong evidence suggested a positive association between circadian disruption and breast cancer risk (relative risk 1.14, 95% confidence interval 1.08 to 1.21). However, the association with short sleep duration (<7 hours a night) in seven contributing studies was much less conclusive (0.96, 0.86 to 1.06), and no dose-response association with sleep deficiency was observed.^6^ Findings from other meta-analyses have been conflicting, with two showing no conclusive evidence that sleep duration is associated with breast cancer risk^7^ ^8^ and one showing evidence of an adverse effect of increased sleep duration (>7 hours a night).^9^ Most studies in the meta-analyses, however, have been case-control designs, vulnerable to reverse causation, or cohort studies with a small number of cases. Fewer studies have investigated associations between chronotype and insomnia with breast cancer risk. The Nurses’ Health Study cohort of 72 517 women (1834 breast cancer cases) found no strong evidence of an association with chronotype,^10^ and a prospective study of 33 332 women (862 incident breast cancer cases) in the Nord-Trøndelag Health Study (HUNT) found no strong evidence of an association with individual insomnia symptoms, although there was evidence of some excess risk among participants with multiple insomnia problems.^11^ Studies have tended to rely on self report of sleep exposures, meaning associations could be biased by measurement error and by residual or unmeasured confounding, making causal inference challenging.

Mendelian randomisation (MR) uses genetic variants that are robustly associated with potentially modifiable risk factors to explore causal effects on outcomes.^12^ ^13^ ^14^ This method is less susceptible to measurement error, confounding, and reverse causation than conventional multivariable regression approaches, provided certain assumptions are met. These are that the genetic variants are robustly associated with the exposure of interest, are not associated with confounders of the exposure-outcome relation, and do not influence the outcome through pathways other than the exposure of interest. Genetic variants robustly associated with chronotype, sleep duration, and insomnia symptoms have recently been identified in large genome-wide association studies (GWAS) with sample sizes of around 50 000 to more than one million.^15^ ^16^ ^17^ ^18^ ^19^ ^20^ ^21^ ^22^ ^23^ Findings from those GWAS have confirmed the role of several core circadian genes influencing sleep traits, and identified genetic variants with no previously known circadian role.^24^ These genetic variants have been used in two sample MR and provided some evidence that longer sleep has a causal effect on schizophrenia risk,^16^ whereas being a “morning person” is causally associated with a reduced risk of schizophrenia and depression,^15^ and insomnia is causally associated with an increased risk of type 2 diabetes, higher body mass index (BMI), coronary heart disease, and several psychiatric traits.^17^ ^23^ In our study we used MR to explore the causal effect of sleep traits on breast cancer risk.

We used genetic variants robustly associated with chronotype, sleep duration, and insomnia symptoms identified in three recent UK Biobank GWAS^15^ ^16^ ^17^ to investigate whether these sleep traits have a causal effect on breast cancer risk. To do this, we performed a one sample MR analysis using data from UK Biobank, from which estimates were compared with conventional observational multivariable regression results in the same study, as well as a two sample MR analysis using data from the Breast Cancer Association Consortium (BCAC).^25^ Furthermore, we aimed to assess the extent to which findings were robust to potential pleiotropy and supported by genetic variants associated with accelerometer derived measures of chronotype (sleep midpoint timing of the least active five hours of the day), sleep duration, and sleep fragmentation (number of nocturnal sleep episodes).

## Methods

### Multivariable regression and one sample MR analysis

#### Study participants

We used data on women from the UK Biobank, which recruited more than 500 000 participants (55% women) out of 9.2 million eligible adults aged between 40 and 70 years in the UK who were invited to participate (5.5% response rate).^26^ The study protocol is available online (www.ukbiobank.ac.uk/wp-content/uploads/2011/11/UK-Biobank-Protocol.pdf) and more details are published elsewhere.^27^ At recruitment the participants gave informed consent to participate and be followed-up. Overall, 503 317 participants consented to join the study cohort and visited an assessment centre. Information on sleep traits (chronotype, sleep duration, and insomnia symptoms), breast cancer status (prevalent and incident cases with up to nine years of follow-up), relevant confounding factors, and genetic variants are available in UK Biobank.

#### Sleep traits

At baseline assessment, conducted in one of 22 UK Biobank assessment centres between 2006 and 2010, participants completed a touchscreen questionnaire, which included questions about sociodemographic status, lifestyle and environment, early life and family history, health and medical history, and psychosocial factors. Participants were asked about their chronotype (morning or evening preference), average sleep duration, and insomnia symptoms.

Chronotype (morning or evening preference) was assessed in the question “Do you consider yourself to be?” with one of six possible answers: “Definitely a ‘morning’ person,” “More a ‘morning’ than ‘evening’ person,” “More an ‘evening’ than a ‘morning’ person,” “Definitely an ‘evening’ person,” “Do not know,” or “Prefer not to answer.” We derived a five level ordinal variable for chronotype where “Definitely a ‘morning’ person,” “More a ‘morning’ than ‘evening’ person,” “More an ‘evening’ than a ‘morning’ person,” “Definitely an ‘evening’ person,” “Do not know,” or “Prefer not to answer” were coded as 2, 1, −1, −2, 0, and missing, respectively. Sleep duration was assessed by asking: “About how many hours sleep do you get in every 24 hours? (please include naps).” The answer could only contain integer values. Binary variables for short sleep duration (<7 hours *v* 7-8 hours) and long sleep duration (>8 hours *v* 7-8 hours) were also derived. To assess insomnia symptoms, participants were asked: “Do you have trouble falling asleep at night or do you wake up in the middle of the night?” with responses “Never/rarely,” “Sometimes,” “Usually,” or “Prefer not to answer.” Those who responded “Prefer not to answer” were set to missing. We derived a three level ordinal variable for insomnia symptoms where “Never/rarely,” “Sometimes,” and “Usually” were coded as 0, 1, and 2, respectively.

#### Breast cancer

Participants were followed through record linkage to the National Health Service central registers, which provide information on cancer registrations, using ICD-9 and ICD-10 (international classification of diseases, ninth and 10th revisions, respectively) codes and cancer deaths. The endpoints in these analyses were first diagnosis of invasive breast cancer (ICD-10 C50, ICD-9 174), or breast cancer listed as the underlying cause of death on the death certificate for women who died during follow-up but were not captured by the cancer registers. We excluded all women with any other cancer diagnosis from the analysis. At the time of analysis, mortality data were available up to February 2016 and cancer registry data up to April 2015. Prevalent cases were defined as women with a diagnosis of breast cancer before date of recruitment to the UK Biobank. Incident cases were defined as women with a diagnosis of breast cancer or dying from it during follow-up.

#### Confounders

We considered several factors to be potential confounders of the association between sleep traits and breast cancer risk: education, body mass index (BMI), alcohol intake, smoking, strenuous physical activity, family history of breast cancer, age at menarche, parity, use of oral contraceptives, menopause status, and hormone replacement therapy.

BMI was derived from weight and height measured when participants attended the initial assessment centre, whereas information on other potential confounders was obtained from questionnaire responses completed at baseline (see methods in supplementary file). Additional information extracted from the initial assessment visit included centre of initial assessment visit, age at recruitment derived from date of birth, and date of attending assessment centre. Participants who were employed were also asked whether their current job involved night shifts: never/rarely, sometimes, usually, or always.

#### Genetic variants

The full data release in UK Biobank contains the cohort of successfully genotyped people (n=488 377). A total of 49 979 people were genotyped using the UK BiLEVE genotyping chip and 438 398 using the UK Biobank axiom genotyping chip. Pre-imputation quality control, phasing, and imputation of the UK Biobank genetic data have been described elsewhere.^28^

In the MR analysis, we used a total of 341 single nucleotide polymorphisms (SNPs) associated with chronotype,^15^ 91 SNPs associated with continuous sleep duration,^16^ and 57 SNPs associated with insomnia symptoms^17^ (see supplementary file, tables 1-3). These genetic variants were derived from self report and confirmed with objective sleep assessment and in independent cohorts.^15^ ^16^ ^17^

#### Multivariable regression analysis

We carried out separate multivariable Cox regression between chronotype, insomnia symptoms, and sleep duration and incident breast cancer to investigate prospective associations between these sleep traits and to minimise the likelihood of reverse causality in observational associations. To minimise the role of confounding, we adjusted analyses for age, assessment centre, and the top 40 genetic principal components (obtained from principal components analysis (PCA) to detect and quantify the genetic structure of populations). A second model additionally adjusted for education, BMI, alcohol intake, smoking, strenuous physical activity, family history of breast cancer, age at menarche, parity, menopause status, use of oral contraceptives, and hormone replacement therapy. 

### One sample MR analysis

For one sample MR, the genetic variants used were extracted genotypes from the UK Biobank imputation dataset (imputed to the Haplotype Reference Consortium reference panel), which performed extensive quality control including exclusion of most third degree or closer relatives from a genetic kinship analysis, as well as those who were not classified as white British based on questionnaire and PCA^29^ (see methods in supplementary file). Unweighted allele scores were generated as the total number of sleep trait increasing alleles (morning preference alleles from chronotype) present in the genotype of each participant.

A two stage method was implemented to give a population average causal hazard ratio. The first stage model consisted of a regression of the sleep trait (chronotype, sleep duration, and insomnia symptoms) on the allele score and the second stage model consisted of a Cox regression of breast cancer status on the fitted values from the first stage regression, with adjustment for age at recruitment, assessment centre, 40 genetic principal components, and genotyping chip in both stages. 

#### Sensitivity analyses

To check the proportional hazards assumption, we used Pearson correlations to test Schoenfeld residuals from both multivariable Cox regression and one sample MR Cox regression models for an association with follow-up time.

To assess the specificity of our findings to breast cancer, we performed multivariable regression and one sample MR analysis to assess the causal effect of the sleep traits on other cancer diagnoses and on all cause mortality.

We also performed MR analysis using only those genetic variants that replicated at Bonferroni significance in a large independent dataset for chronotype^15^ (242 variants in 23andMe, n=240 098, highlighted in supplementary file, table 1) to evaluate the potential impact of winner’s curse (ie, overestimation of genetic effects in the initial study), which can bias causal estimates in MR analysis. Given the relatively small sample size of replication datasets for sleep duration (CHARGE Consortium, n=47 180)^16^ and insomnia (HUNT, n=62 533),^17^ few SNPs independently replicated at Bonferroni significance to serve as sufficiently strong instruments for this sensitivity analysis.

To test the MR assumption that genetic variants should not be associated with confounders of the exposure-outcome relation, we investigated associations between the allele scores and potential confounders in UK Biobank. We then performed one sample MR analysis adjusted for any potential confounders found to be strongly associated with the allele scores (beyond a Bonferroni significance threshold of P<1.39×10^−3^) as a further sensitivity analysis.

We also conducted both multivariable regression and one sample MR using all breast cancer cases (incident and prevalent) in a logistic regression analysis in UK Biobank, and performed sensitivity analysis removing participants who reported currently working night shifts (sometimes, usually, or always).

### Two sample MR analysis

We conducted a two sample MR analysis of sleep traits on breast cancer risk using female specific estimates of the associations between the genetic instruments and sleep traits identified in the respective GWAS^15^ ^16^ ^17^ in UK Biobank (sample 1) (see supplementary file, tables 1-3), and estimates of the associations between the genetic instruments and breast cancer from a large scale GWAS of breast cancer (BCAC) (sample 2).

GWAS of chronotype (five level ordinal variable), sleep duration (continuous variable), and insomnia symptoms (three level ordinal variable) were performed among women of European ancestry (n=241 350 - 245 767) in the UK Biobank. This was done using BOLT-LMM^30^ linear mixed models and an additive genetic model adjusted for age, sex, 10 genetic principal components, genotyping array, and genetic correlation matrix, as was done previously.^15^ ^16^ ^17^

The GWAS of breast cancer involved 122 977 women with the disease (oestrogen receptor positive and oestrogen receptor negative combined) and 105 974 controls of European ancestry from BCAC.^25^ BCAC summary data were based on imputation to the 1000 Genomes Project Phase 3 reference panel. To explore potential heterogeneity by breast cancer subtype, we also investigated the causal effect of the sleep traits on breast cancer stratified by oestrogen receptor status, using genetic association data from 69 501 oestrogen positive and 21 468 oestrogen negative cases within BCAC.^25^

Two sample MR analyses were conducted using “TwoSampleMR,” an R package for such analyses,^31^ which was first used to extract the SNPs being used to instrument the exposure (here the sleep trait of interest) from the outcome GWAS (here breast cancer in BCAC). If a SNP was unavailable in the breast cancer GWAS summary statistics, we identified proxy SNPs with a minimum linkage disequilibrium (LD) r^2^=0.8. We then performed harmonisation of the direction of effects between exposure and outcome associations, where palindromic SNPs were aligned when minor allele frequencies were less than 0.3, or they were otherwise excluded. We then used an inverse variance weighted method to meta-analyse the SNP specific Wald estimates (SNP outcome estimate divided by SNP exposure estimate) using random effects, to obtain an estimate for the causal effect of the sleep trait on breast cancer risk.

#### Sensitivity analyses

The inverse variance weighted random effects method will return an unbiased estimate in the absence of horizontal pleiotropy, or when horizontal pleiotropy is balanced.^32^ To account for directional pleiotropy, we compared results with three other MR methods, which each makes different assumptions about this: MR Egger,^33^ weighted median,^34^ and weighted mode,^35^ and therefore a consistent effect across multiple methods strengthens causal evidence.

To further detect and correct obtained causal estimates for potential violation of the MR assumptions,^32^ we performed RadialMR^36^ in the two sample analyses to identify outliers with the most weight in the MR analysis and the largest contribution to Cochran’s Q statistic for heterogeneity, which may then be removed and the data reanalysed. Radial MR analysis was conducted using modified second order weights and an α level of 0.05 divided by the number of SNPs being used to instrument the exposure. For the outliers identified, we also assessed their potential pleiotropic role by performing a phenome-wide association study (PheWAS) approach^37^ to investigate the associations between the SNPs and all available traits in the MR-Base PheWAS database (http://phewas.mrbase.org/).

To evaluate the potential impact of winner’s curse, we performed two sample MR analysis using 242 genetic variants that replicated at Bonferroni significance in a large independent dataset for chronotype^15^ (23andMe, n=240 098, highlighted in supplementary file, table 1). We also carried out further MR analysis using robust adjusted profile scores, which provide an unbiased causal estimate in the presence of weak instruments.^38^

Given potential non-linear associations between sleep duration and breast cancer risk,^9^ we also used data on 27 SNPs associated with short sleep (<7 hours *v* 7-8 hours) and eight SNPs associated with long sleep (>8 hours *v* 7-8 hours)^16^ in two sample MR analysis (see supplementary file, tables 4 and 5). Causal effect estimates (ie, odds ratios for breast cancer) were rescaled to be interpreted for each doubling of genetic liability for short or long sleep, as recommended elsewhere.^39^

Finally, we performed two sample MR using genetic variants robustly associated with accelerometer derived sleep traits in UK Biobank, to be compared with causal estimates obtained using genetic variants associated with self reported traits. For this we used genetic variants identified in GWAS in relation to three accelerometer based measures: timing of the least active five hours (L5 timing) (6 SNPs), nocturnal sleep duration (11 SNPs), and number of nocturnal sleep episodes (21 SNPs) in up to 85 205 participants, as previously described^40^ (see supplementary file, tables 6-8). Also see the methods section in the supplementary file for more details about how accelerometer sleep traits were derived. Effect estimates represented an hour earlier L5 timing (correlated positively with and to be compared with the self reported chronotype measure of increased morning preference), an hour increase of nocturnal sleep duration (to be compared with self reported sleep duration), and a unit increase in the number of nocturnal sleep episodes (to be compared with self reported insomnia symptoms).

All analyses were conducted using Stata (version 15) and R (version 3.4.1).

### Patient and public involvement

The current research was not informed by patient and public involvement because it used secondary data. However, future research following on from our findings should be guided by patient and public opinions.

No patients were involved in setting the research question or the outcome measures, nor were they involved in developing plans for design or implementation of the study. No patients were asked to advise on interpretation or writing up of results. The results of the research will be disseminated to study participants on request, and to stakeholders and the broader public as relevant. 

## Results

### Baseline characteristics

Of the 180 216 women in the UK Biobank who had been successfully genotyped and passed the genetic quality control, and after excluding 23 368 who had a diagnosis of other types of cancer, 7784/156 868 (4.9%) had received an exclusive diagnosis of breast cancer. Of these, 5036/156 868 (3.2%) were defined as prevalent cases and 2740/156 868 (1.7%) developed incident breast cancer over a median follow-up of 2.98 years.

Women with a breast cancer diagnosis (prevalent or incident) were more likely to be older, have a higher BMI, be less physically active, have had an earlier age at menarche, be postmenopausal, have ever used hormone replacement therapy, have a family history of breast cancer, and be nulliparous. They were less likely to be never smokers, work night shifts, and have ever used oral contraceptives (table 1) compared with women without a breast cancer diagnosis. No strong difference was found in education level between women with and without breast cancer, in line with previous findings,^41^ as well as no clear difference in relation to alcohol intake.

**Table 1:** Baseline characteristics of women who had and had not developed breast cancer by date of censoring in UK Biobank. Values are numbers (percentages) unless stated otherwise

Characteristic	No breast cancer diagnosis (n=149 064)	Breast cancer diagnosis (n=7784)
Mean (SD) age at recruitment (years); No	56.2 (7.9); n=149 064	58.9 (7.0); n=7784
Mean (SD) body mass index; No	27.0 (5.1); n=148 617	27.2 (4.9); n=7764
Mean (SD) age at menarche; No	13.0 (1.6); n=144 845	12.8 (1.6); n=7576
Mean (SD) days/week strenuous physical activity; No	1.7 (1.8); n=141 387	1.5 (1.8); n=7325
Education:		
Degree	65 381 (44.3)	3337 (43.3)
No degree	89 383 (55.8)	4376 (56.7)
Smoking:		
Never	90 072 (60.6)	4445 (57.4)
Former	46 374 (31.2)	2700 (34.8)
Current	12 131 (8.2)	604 (7.8)
Alcohol use:		
Never	6254 (4.2)	343 (4.4)
Former	5208 (3.5)	287 (3.7)
Current	137 465 (92.3)	7145 (91.9)
Family history of breast cancer:		
Yes	9221 (6.2)	526 (6.8)
No	139 843 (93.8)	7258 (93.2)
Parity:		
0	27 508 (18.5)	1489 (19.1)
≥1	121 479 (82.5)	6290 (80.9)
Oral contraceptive use:		
Yes	123 688 (83.1)	6193 (79.7)
No	25 117 (16.9)	1580 (20.3)
Menopause:		
Yes	89 397 (60.0)	5721 (73.6)
No	36 272 (24.4)	787 (10.1)
Not sure	23 277 (15.6)	1266 (16.3)
Hormone replacement therapy:		
Yes	57 038 (38.4)	3142 (40.5)
No	91 682 (61.7)	4620 (59.5)
Shift work:		
Night	4815 (5.8)	158 (4.6)
Other	6763 (8.1)	282 (8.2)
None	71 790 (86.1)	3014 (87.3)

### Multivariable analysis

In multivariable Cox regression analysis, an inverse association was observed between morning preference and risk of breast cancer, which remained similar in the fully adjusted model (hazard ratio 0.95, 95% confidence interval 0.93 to 0.98 per category increase) but there was no clear association between sleep duration and insomnia symptoms with risk of breast cancer (table 2). The proportional hazards assumption held for all the multivariable Cox regression analyses (see supplementary file, table 9). The inverse association with morning preference was not observed for other cancer diagnoses (hazard ratio 1.00, 95% confidence interval 0.99 to 1.02 per category increase) (see supplementary file, table 10), although it was evident in multivariable Cox regression analysis of all cause mortality (0.95, 0.93 to 0.97 per category increase) (see supplementary file, table 11). Associations with sleep duration and insomnia were also observed in relation to these other outcomes (see supplementary file, tables 10 and 11).

**Table 2:** Multivariable and mendelian randomisation Cox regression analysis for risk of breast cancer associated with sleep traits

Sleep trait	Basic model*				Fully adjusted model†				Mendelian randomisation analysis‡		
	No (incident cases)	Hazard ratio (95% CI)	P value		No (incident cases)	Hazard ratio (95% CI)	P value		No (incident cases)	Hazard ratio (95% CI)	P value
Chronotype (per category increase§)	151 421 (2732)	0.94 (0.92 to 0.97)	<0.001		138 529 (2500)	0.95 (0.93 to 0.98)	0.002		151 421 (2732)	0.85 (0.70 to 1.03)	0.10
Sleep duration (per hour increase)	150 845 (2723)	1.01 (0.98 to 1.05)	0.55		138 228 (2495)	1.00 (0.96 to 1.04)	0.98		150 845 (2723)	1.06 (0.70 to 1.59)	0.78
Insomnia symptoms (per category increase¶)	149 005 (2740)	1.02 (0.97 to 1.08)	0.44		138 771 (2505)	1.02 (0.97 to 1.08)	0.44		149 005 (2740)	1.37 (0.59 to 3.20)	0.47

* Adjusted for age, assessment centre, and top 40 genetic principal components.

† Adjusted for age, assessment centre, top 40 genetic principal components, degree status, body mass index, alcohol intake, smoking, strenuous physical activity, family history of breast cancer, parity, age at menarche, menopause status, use of oral contraceptives, and hormone replacement therapy.

‡ Adjusted for age, assessment centre, top 40 genetic principal components, and genotyping chip.

§ From definite evening, intermediate evening, don’t know, intermediate morning, and definite morning.

¶ From none, some, and frequent.

When incident and prevalent cases were combined and associations investigated in a logistic regression framework, evidence was consistent for an inverse association between morning preference and breast cancer risk (odds ratio 0.96, 95% confidence interval 0.94 to 0.98), as well a positive association between both sleep duration (1.02, 1.00 to 1.05 per hour increase) and insomnia symptoms (1.11, 1.07 to 1.15 per category increase) with breast cancer risk, potentially reflecting reverse causation (see supplementary file, table 12). Cox regression estimates were similar after excluding participants who reported working night shifts (see supplementary file, table 13).

### One sample MR analysis

Among UK Biobank female participants, allele scores explained 2.3% of the variance in chronotype, 0.7% of the variance in sleep duration, and 0.4% of the variance in insomnia symptoms (table 3). Some evidence suggested a protective effect of morning preference on breast cancer risk (hazard ratio 0.85, 95% confidence interval 0.70 to 1.03 per category increase) and weaker evidence for an adverse effect of increased sleep duration (1.06, 0.70 to 1.59 per hour increase) and insomnia symptoms (1.37, 0.59 to 3.20 per category increase) (table 2), albeit imprecisely estimated (wide confidence intervals). The proportional hazards assumption held for all the one sample MR Cox regression analyses (see supplementary file, table 9). The protective effect of morning preference was not supported by MR analysis for other cancer diagnoses (1.05, 0.93 to 1.17 per category increase) (see supplementary file, table 10) or all cause mortality (1.15, 0.97 to 1.35 per category increase) (see supplementary file, table 11), although evidence suggested an adverse effect of insomnia on risk of other cancers (1.55, 0.94 to 2.55 per category increase) (see supplementary file, table 10).

**Table 3:** Allele scores for sleep traits in UK Biobank

Sleep trait	No (incident cases)	Mean (SD) No of increasing alleles	Association of allele score with sleep trait*			
			Coefficient (SE)	P value	R^2^	F statistic
Chronotype (morning preference)	151 421 (2732)	336 (11.6)	0.017 (0.0003)	<0.001	0.0229	3666
Sleep duration	150 845 (2723)	90 (5.9)	0.015 (0.0004)	<0.001	0.0072	1127
Insomnia symptoms	149 005 (2740)	56 (4.9)	0.012 (0.0002)	<0.001	0.0041	639

* Adjusted for age, assessment centre, 40 principal components, and genotyping chip.

When using only the genetic variants that replicated in an independent dataset (242 variants in 23andMe) for chronotype, estimates of effect on breast cancer risk were similar (0.89, 0.71 to 1.12 per category increase); although with wider confidence intervals given that the replicated variants explained less of the variance in chronotype (1.6%) (see supplementary file, table 14).

Although most of the confounding factors were not associated with the sleep trait allele scores in UK Biobank, after accounting for multiple testing the chronotype allele score was associated with parity and vigorous activity; the sleep duration allele score was associated with age at menarche and BMI, and the insomnia allele score was associated with using hormone replacement therapy and age at menarche (see supplementary file, table 15). Further sensitivity analysis was undertaken adjusting for these potential confounders in the one sample MR analysis, and effect estimates were consistent (see supplementary file, table 16).

Findings of a protective effect of morning preference were supported in analysis of all breast cancer cases (incident and prevalent) in logistic regression. Evidence for sleep duration and insomnia symptoms was weaker, although both had effect estimates in the positive direction (see supplementary file, table 12). In analyses excluding women who reported working night shifts, findings were also consistent with the main results from Cox regression (see supplementary file, table 13).

### Two sample MR analysis

**Fig 1:**
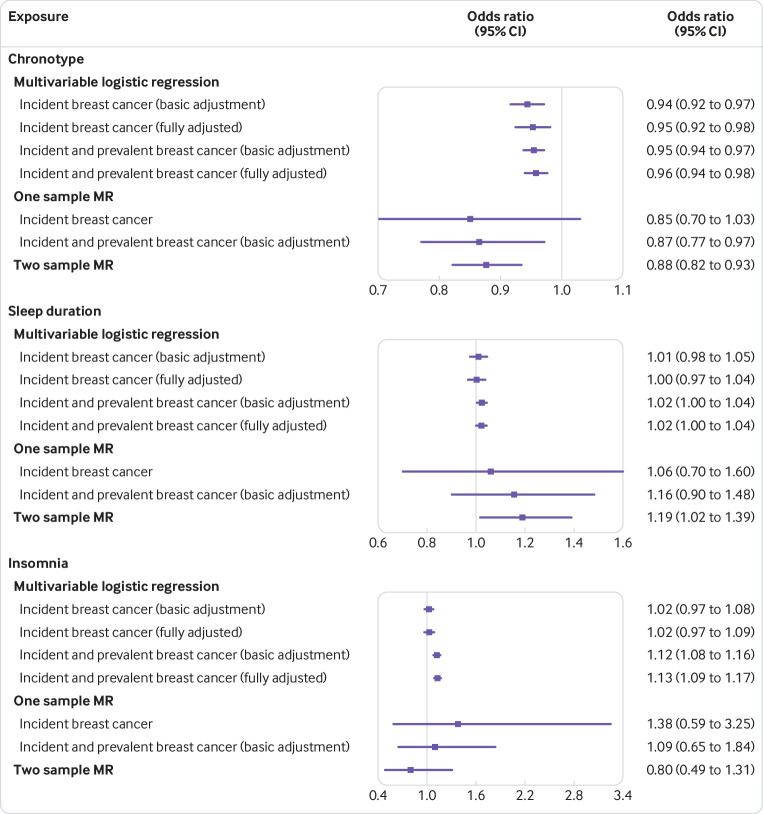
Forest plot of multivariable and mendelian randomisation (MR) estimates for association between sleep traits and breast cancer risk. Odds ratios are per category increase in chronotype (from definite evening, intermediate evening, neither, intermediate morning, definite morning), per hour increase in sleep duration, and per category increase in insomnia risk (from no, some, and frequent insomnia symptoms). Odds ratios rather than hazard ratios for incident breast cancer are shown for multivariable and one sample MR analysis to compare estimates across methods

After harmonisation of the SNP effects in the two summary datasets (UK Biobank and BCAC), 305 SNPs were used to instrument chronotype, 82 SNPs were used to instrument sleep duration, and 50 SNPs were used to instrument insomnia symptoms. This included three proxy SNPs (r^2^≥0.8) for chronotype (rs376957969 for rs111867612, rs1871516 for rs4550782, and rs6583802 for rs61875203). Two sample MR supported the findings of a protective effect of morning preference (inverse variance weighted odds ratio 0.88, 95% confidence interval 0.82 to 0.93 per category increase) (see supplementary file, table 17 and figure 1) as well as an adverse effect of increased sleep duration (1.19, 1.02, 1.39 per hour increase) on breast cancer risk (see supplementary file, table 17 and figure 2). Little evidence for a causal effect of insomnia symptoms was observed (0.93, 0.49, 1.76 per category increase) (see supplementary file, table 17 and figure 3). Figure 1 shows the inverse variance weighted estimates for chronotype, sleep duration, and insomnia symptoms from two sample MR compared with multivariable and one sample MR approaches in UK Biobank. Findings were similar when stratified by oestrogen receptor positive and oestrogen receptor negative breast cancer (see supplementary file, table 17).

Effect estimates were broadly consistent between the inverse variance weighted method and the pleiotropy robust methods applied (MR Egger, weighted median, and weighted mode) in two sample MR (see supplementary file, table 17 and figures 1-3). Furthermore, the MR Egger test of directional pleiotropy was consistent with the null for all analyses (see supplementary file, table 18).

Evidence for heterogeneity in causal effects for most of the models (see supplementary file, table 19) could still indicate potential violations of the MR assumptions. We used radial plots to aid in the detection of outlying variants. Radial MR analysis identified six outliers for chronotype, three for sleep duration, and two for insomnia symptoms in both inverse variance weighted and MR Egger (see supplementary file, table 20 and figures 4-6). The pleiotropic effect of many of these outliers was indicated in a PheWAS of the SNPs on all existing traits in the MR-Base database (see supplementary file, figure 7). With removal of outliers, inverse variance weighted and MR Egger effect estimates were largely unchanged (see supplementary file, table 21).

Effect estimates for the causal effect of chronotype on breast cancer risk were consistent when using the 242 genetic variants associated with chronotype, which replicated at Bonferroni significance in 23andMe,^15^ indicating that winner’s curse is unlikely to have substantially biased effect estimates (see supplementary file, table 22). MR robust adjusted profile scores, which provide unbiased estimates in the presence of weak instruments, provided similar causal estimates to the main MR analysis (see supplementary file, table 23).

Findings of an adverse effect of increased sleep duration on breast cancer risk were supported using genetic variants specifically associated with short and long sleep duration, with evidence for a protective effect of short sleep duration on breast cancer (inverse variance weighted odds ratio 0.92, 95% confidence interval 0.86 to 0.99 per doubling of genetic liability for short sleep duration) and adverse effect of long sleep duration (1.24, 0.96 to 1.60 per doubling of genetic liability for long sleep duration) (see supplementary file, table 24).

Finally, we performed two sample MR using genetic variants robustly associated with accelerometer derived sleep traits in UK Biobank, to be compared with causal estimates obtained using genetic variants associated with self reported traits. Supplementary table 25 shows the genetic correlations between these traits. Using genetic variants robustly associated with accelerometer derived sleep traits in UK Biobank, we found no clear evidence of association with L5 timing measured objectively (1.04, 0.78 to 1.38 per hour decrease) (see supplementary file, table 26 and figure 8). However, an adverse effect of increased sleep duration was supported using estimates from objectively measured sleep duration (1.16, 1.02 to 1.32 per hour increase) (see supplementary file, table 26 and figure 9) and there was some evidence for a causal effect of increased fragmentation on breast cancer risk (1.14, 1.00 to 1.30 per sleep episode) (see supplementary file, table 26 and figure 10). Given the limited availability of SNPs being used to proxy for L5 timing to evaluate its causal role on breast cancer, and given the strong association found between chronotype and L5 timing (see supplementary file, table 25),^15^ we performed a further MR analysis using the 305 chronotype variants with SNP exposure effect estimates taken from the GWAS of L5 timing, to also evaluate the causal effect of L5 timing (see supplementary file, table 27). This analysis revealed some evidence for an association with L5 timing and risk of breast cancer in the inverse variance weighted analysis (0.86, 0.78 to 0.95), although this estimate was not consistent across the pleiotropy robust methods, which were more consistent with the null.

## Discussion

Mendelian randomisation (MR) uses genetic variation to investigate causal relations between potentially modifiable risk factors and health outcomes. In this study we compared observational estimates from multivariable regression with those from MR analyses to make inferences about the likely causal effects of three sleep traits on breast cancer risk. 

In multivariable regression analysis using data on breast cancer incidence in the UK Biobank study, morning preference was inversely associated with breast cancer, whereas there was little evidence for an association with sleep duration and insomnia. Using genetic variants associated with chronotype, sleep duration, and insomnia symptoms, one sample MR analysis in UK Biobank provided some evidence for a protective effect of morning preference but imprecise estimates for sleep duration and insomnia. Findings for a protective effect of morning preference and adverse effect of increased sleep duration on breast cancer (both oestrogen receptor positive and oestrogen receptor negative) were supported by two sample MR using data from the Breast Cancer Association Consortium (BCAC), whereas there was inconsistent evidence for insomnia symptoms. Results were largely robust to sensitivity analyses accounting for horizontal pleiotropy.

### Comparison with other studies

Previous studies have found an enrichment of circadian pathway genetic variants in breast cancer.^25^ ^42^ Nonetheless, these studies did not directly implicate modifiable sleep traits by which risk of breast cancer could be minimised and did not attempt to separate the effects of the genetic variants on breast cancer risk through circadian disruption from pleiotropic pathways.

Findings of an adverse effect of evening preference on breast cancer risk in all analyses performed go some way to supporting hypotheses around carcinogenic light-at-night 4 and findings of increased risk among night shift workers who might be exposed to artificial light at night.^1^ In particular, the specificity of the causal effect of chronotype on breast cancer, which was not observed in relation to other cancers or all cause mortality, is consistent with the hormonal mechanisms implicated in the light-at-night hypothesis. However, findings when using an objective measure of chronotype (the least active five hours (L5 timing)) did not reveal the same adverse effect. Although this last analysis might be limited by the number and strength of the genetic variants used to instrument L5 timing, the lack of consistency in estimates draws to question the mechanisms by which morning or evening preference (rather than actual activity) influences breast cancer risk. Further analysis using the single nucleotide polymorphisms (SNPs) identified in relation to chronotype as instruments for L5 timing were consistent with a protective effect of morning preference, suggesting a protective effect of activity as well as reported preference, but as the pleiotropy robust tests were not consistent, more work is needed to distinguish the causal effect of morning preference from activity—for example, with the use of multivariable MR methods.^43^

Evidence for an adverse effect of increased sleep duration on breast cancer risk contrasts with the observational findings in UK Biobank as well as much of the literature on circadian disruption and breast cancer risk,^6^ and unlike our findings for chronotype are not aligned with the light at night hypothesis. However, recent studies implicate longer sleep duration as a risk factor for breast cancer.^9^ Given previous reports of a J-shaped relation between sleep duration and breast cancer risk,^9^ as well as investigating sleep duration as a continuous variable, we also investigated the causal effects of both short and long sleep duration to investigate non-linear effects. In line with our main findings, we found evidence for a protective effect of short sleep duration and adverse effect of long sleep duration on breast cancer risk. Furthermore, using genetic variants associated with accelerometer derived nocturnal sleep duration, we found evidence for an adverse effect of sleep duration with a similar magnitude of effect.

Overall, we found inconsistent evidence about the causal effect of insomnia symptoms on breast cancer risk in multivariable and MR analyses. A previous study of incident breast cancer in the Nord-Trøndelag Health Study (HUNT) revealed no strong evidence of an association with individual insomnia symptoms,^11^ although people with multiple insomnia problems were found to be at increased risk. In our analysis, insomnia was defined based on self report of either difficulty initiating sleep or waking in the night. Further work is therefore required to investigate individual symptoms of insomnia on breast cancer risk, and the potential cumulative effect. Interestingly, MR analysis provided some evidence for adverse causal effect of accelerometer derived number of nocturnal sleep episodes on breast cancer risk.

### Strengths and limitations of this study

Key strengths of the study are the integration of multiple approaches to assess the causal effect of sleep traits on breast cancer, the inclusion of data from two large epidemiological resources—UK Biobank and BCAC—as well as use of data derived from both self reported and objectively assessed measures of sleep. Furthermore, for MR analysis we used the largest number of SNPs identified in the genome-wide association studies (GWAS) literature, with full summary statistics available to obtain strong genetic instruments for MR analysis and to explore potential pleiotropic pathways.

The approaches of multivariable Cox regression of incident cases, multivariable logistic regression of prevalent and incident cases, one and two sample MR, each have different strengths and limitations in terms of key sources of bias (see supplementary file, table 28). In multivariable analysis, attempts were made to mitigate key sources of bias, including confounding and reverse causation, with the use of multivariable Cox regression analysis of incident cases of breast cancer and adjustment for several hypothesised confounders. Nonetheless, residual or unmeasured confounding, selection bias, and measurement error could also have distorted effect estimates. We used MR analysis to minimise the likelihood of bias due to measurement error, confounding, and reverse causation. In addition, we conducted a series of sensitivity analyses to assess the core assumptions that the genetic instruments are strongly associated with the exposures of interest, are not influenced by confounding factors, and do not directly influence the outcome other than through the exposure.

One limitation of this study related to the self reported measures used in multivariable regression analyses and used to identify genetic variants for MR analysis. In particular, the measure of sleep duration might capture time spent napping and the any insomnia variable is really a measure of insomnia symptoms and not necessarily clinical insomnia. However, both these measures have been validated with the use of objective measures from accelerometer data in the UK Biobank and concordance is good, particularly for the effects of the genetic variants identified.^15^ ^16^ ^17^

Another limitation relates to the selection of participants. Analysis in the two large epidemiological studies included here (UK Biobank and BCAC) was restricted to women of European ancestry. Further work is required to investigate whether these findings translate to women in other ancestry groups. Although the UK Biobank represents a large and well characterised epidemiological resource, it is not representative of the UK population owing to low participation.^27^ As well as influencing the generalisability of findings, selection into the study can lead to biased estimates of association through “collider bias.”^44^ To minimise the influence of this, we also used genetic data from a large case-control study of breast cancer (BCAC), and we compared MR effect estimates across these datasets.

In all MR analyses, SNP exposure estimates were obtained from the UK Biobank as this has formed a major component of the GWAS of sleep traits conducted to date.^15^ ^16^ ^17^ ^20^ ^21^ ^23^ This could lead to winner’s curse, when the magnitude of the effect sizes for genetic variants identified within a discovery sample are likely to be larger than in the overall population. In a one sample MR analysis, the impact of winner’s curse of the SNP exposure association can bias causal estimates towards the confounded observational estimate, whereas in two sample MR, winner’s curse can result in bias of the causal estimate towards the null. To minimise the impact of winner’s curse in one sample MR analysis we derived an additional allele score for chronotype composed of SNPs that replicated beyond a Bonferroni correction threshold in an independent study (23andMe).^15^ Similarly, for two sample MR analysis, we used SNP exposure estimates from this replication analysis in sensitivity analyses, and findings were consistent with the main analysis (see supplementary file, tables 14 and 22).

We were unable to apply the same approach to investigate the impact of winner’s curse in the sleep duration and insomnia analysis owing to the relatively small sample size of the replication datasets in those studies, meaning genetic associations could be imprecise. Although we are aware of a large GWAS for insomnia that was conducted using data from both UK Biobank and 23andMe, full summary data for the top SNPs in the replication analysis are not freely available.^23^ We used unweighted allele scores to minimise the contribution of potential weak instruments in the one sample MR analysis. We also applied a robust adjusted profile score method in the two sample MR analysis, which provides unbiased estimates in the presence of weak instruments, and this revealed similar causal estimates for chronotype, sleep duration, and insomnia as in the main analysis.

Although associations between the allele scores and confounders in UK Biobank imply violation of the MR assumption that genetic variants should not be associated with confounding factors, there are several explanations for these findings. Previous MR studies have identified causal effects of sleep traits on reproductive traits, body mass index, and activity levels,^15^ ^16^ ^17^ ^23^ suggesting that these factors might be mediators of the association between sleep traits and breast cancer rather than confounders. Furthermore, some of the genetic variants associated with chronotype and insomnia have been found to be adiposity related loci,^15^ ^16^ implying potential pleiotropic pathways. Nonetheless, we also applied a series of pleiotropy robust MR methods and outlier detection to rigorously explore the possibility that findings of a causal effect of chronotype and sleep duration were not biased as a result of pleiotropy.

As well as attempting to mitigate key sources of bias for each epidemiological approach applied, we also assessed the consistency in estimates between the approaches to provide the best inference about the causal effect of sleep traits on breast cancer. This is aligned with the practice of triangulation, which aims to obtain more reliable answers to research questions through the integration of results from different approaches, where each approach has different sources of potential bias that are unrelated to each other.^45^ ^46^ We also compared estimates based on self reported sleep with the use of genetic variants associated with accelerometer derived measures of sleep,^40^ although we did not use female specific SNP estimates here given the smaller number of participants in UK Biobank with these data.

### Implications of findings

Findings of a protective effect of morning preference on breast cancer risk add to other evidence from MR supporting a possible beneficial effect of morning preference on decreased risk of schizophrenia and depression.^15^ However, whether it is the actual behaviour that poses the health risk or the preference for morning versus evening requires further evaluation. Further work is also required to investigate the impact of circadian misalignment, which can be determined by genetic risk, self reported chronotype, and objectively measured L5 timing. In addition, suggestive evidence for a causal effect of increased sleep duration on breast cancer risk should be investigated further.

### Conclusions

In this study, both multivariable regression and MR analysis were used to provide strong evidence for a causal effect of chronotype on breast cancer risk. Furthermore, some evidence suggested a causal effect of sleep duration on risk of breast cancer, although findings for these traits were less consistent across the different methods applied. However, the biological role of many of the genetic variants used to instrument these traits in MR and mechanistic pathways underlying the observed effects are not well understood. Previously reported pathways between sleep disruption and mammary oncogenesis include immunological, molecular, cellular, neuroendocrine, and metabolic processes.^5^ Further work to uncover these possible mediating processes is required. Nonetheless, these findings have potential implications for influencing sleep habits of the general population to improve health.

**What is already known on this topic**

The World Health Organization’s International Agency for Research on Cancer classifies shift work involving circadian disruption as probably carcinogenic to humans
Much of the literature on breast cancer risk has focused on the potentially adverse effects of night shift work and exposure to light at night, and less into the potential adverse effects of traits such as chronotype (morning or evening preference), sleep duration, and insomnia
Genetic variants robustly associated with chronotype, sleep duration, and insomnia symptoms have recently been identified in large genome-wide association studies

**What this study adds**

This study found consistent evidence for a protective effect of morning preference and suggestive evidence for an adverse effect of increased sleep duration on breast cancer risk
The evidence for insomnia symptoms was inconclusive
These findings have potential implications for influencing sleep habits of the general population to improve health

## Web extra

Extra material supplied by authors

## Notes

Contributors: RCR conceived the study and conducted the main analysis. HSD, SEJ, and JML conducted the female specific genome-wide association studies and assisted with sensitivity analyses. RCR, ELA, and GDS drafted the initial manuscript. All authors assisted with interpretation, commended on drafts of the manuscript, and approved the final version. RCR is the guarantor and attests that all listed authors meet authorship criteria and that no others meeting the criteria have been omitted.

Ethical approval: UK Biobank has received ethical approval from the UK National Health Service’s National Research Ethics Service (ref 11/ NW/0382).

Data sharing: Scripts for the two sample mendelian randomisation analysis are available on GitHub at: https://github.com/rcrichmond/sleep_breastcancer_mr/. For statistical code relating to the individual level data analysis in UK Biobank, please contact the corresponding author at rebecca.richmond@bristol.ac.uk.

Transparency: The lead author (RCR) affirms that this manuscript is an honest, accurate, and transparent account of the study being reported; that no important aspects of the study have been omitted; and that any discrepancies from the study as planned (and, if relevant, registered) have been explained.
