# Supplementary material for: Investigating causal relations between sleep traits and risk of breast cancer in women: mendelian randomisation study
Source: BMJ. 2019 Jun 26;365:l2327. doi: 10.1136/bmj.l2327 (PMC6592406; doi:10.1136/bmj.l2327)
Supplement: Supplementary file 1 — Supplementary information: additional methods, tables, and figures [file ricr048387.ww1.pdf]

Supplementary Figures

Supplementary Figure 1 – Scatter plot of individual SNP-chronotype and SNP-breast cancer associations with overlay of causal estimate from each MR test in two-sample MR analysis

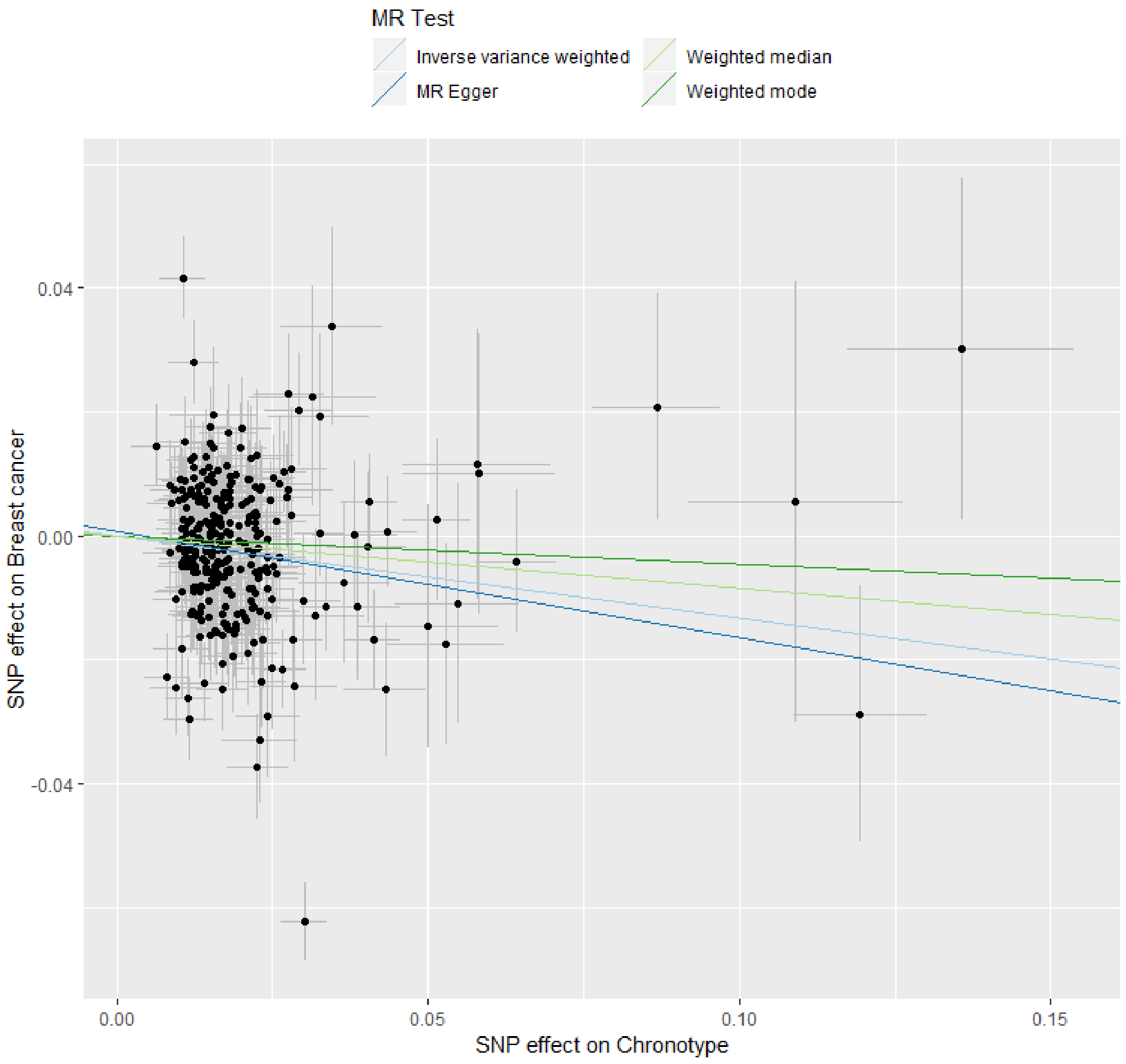

Supplementary Figure 2 – Scatter plot of individual SNP-sleep duration and SNP-breast cancer associations with overlay of causal estimate from each MR test in two-sample MR analysis

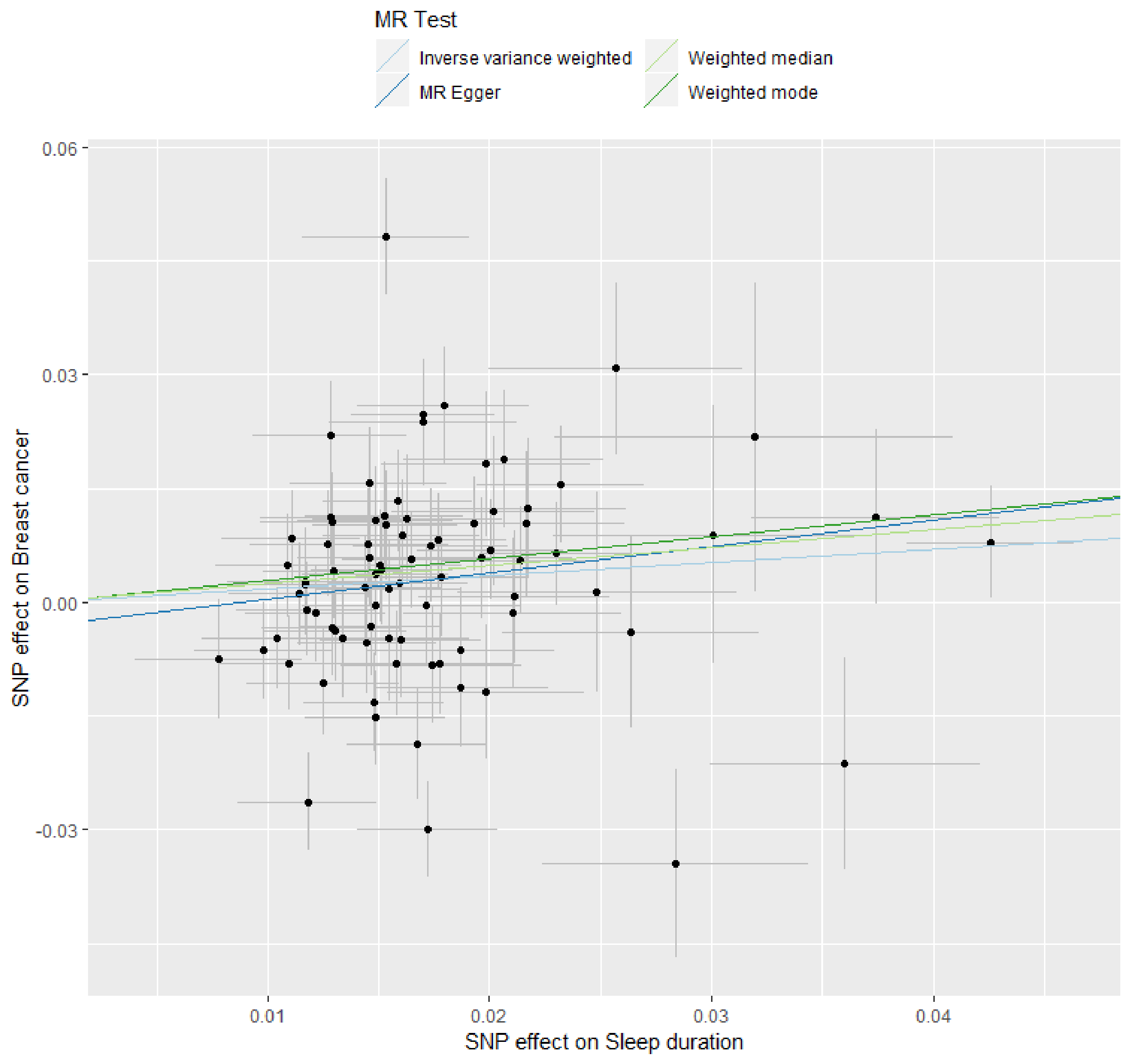

Supplementary Figure 3 – Scatter plot of individual SNP-insomnia and SNP-breast cancer associations with overlay of causal estimate from each MR test in two-sample MR analysis

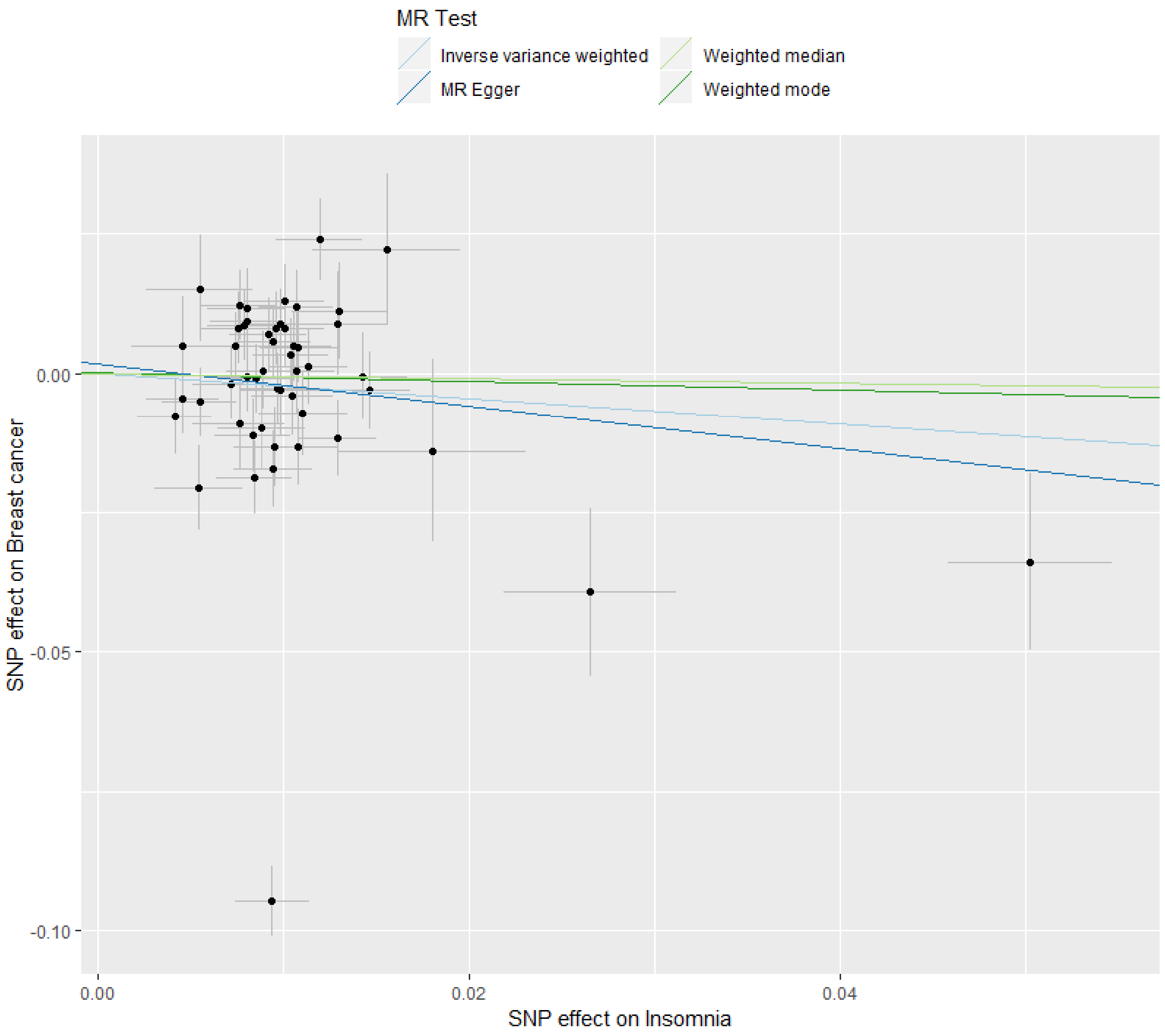

MR Test
Inverse variance weighted
MR Egger
Weighted median
Weighted mode
SNP effect on Breast cancer
0.00
-0.05
-0.10
0.00
0.02
0.04
SNP effect on Insomnia

Supplementary Figure 4 – Radial MR plot for chronotype

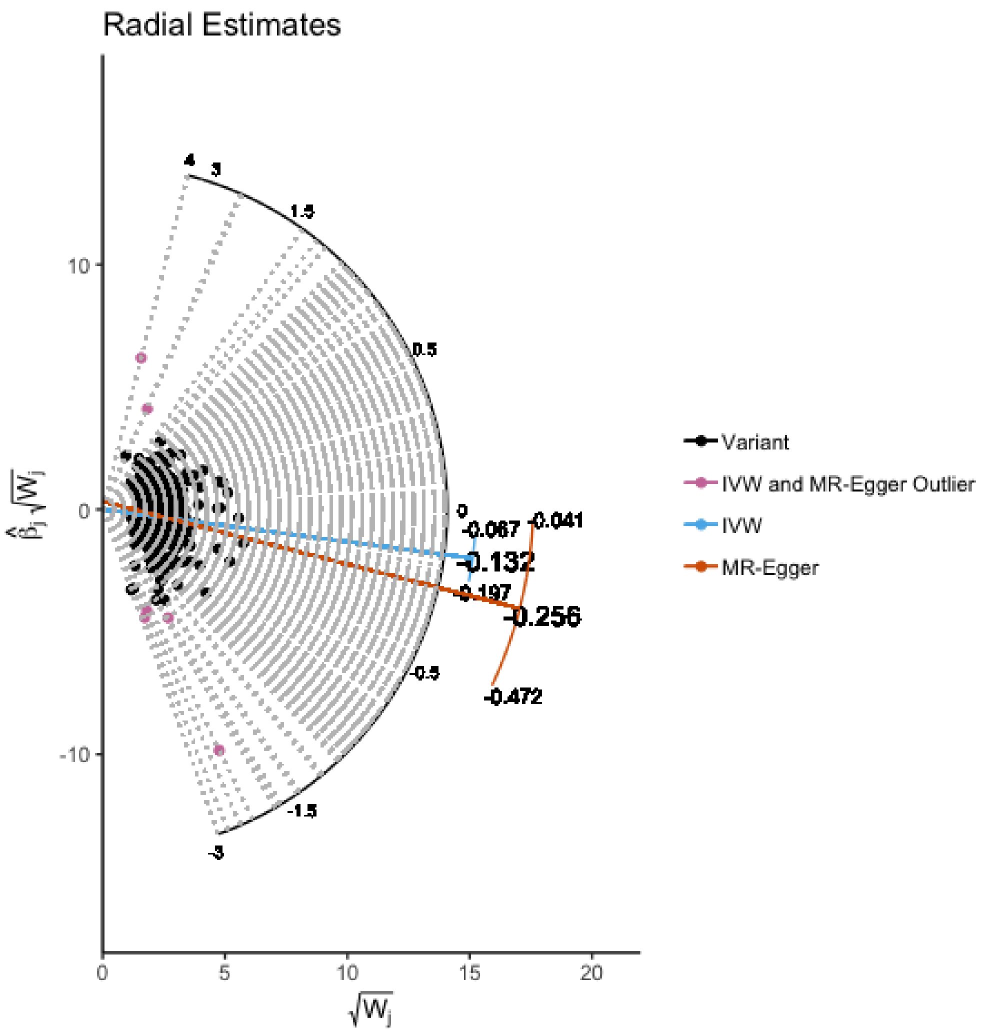

Radial curve displays the ratio estimate for each genetic variant, as well as the overall IVW (in blue) and MR Egger estimate (in orange). Data points with large contributions to Cochran's Q statistic are shown in pink, with an alpha of 0.05/305 = 1.6×10^{-4}.

Supplementary Figure 5 – Radial MR plot for sleep duration

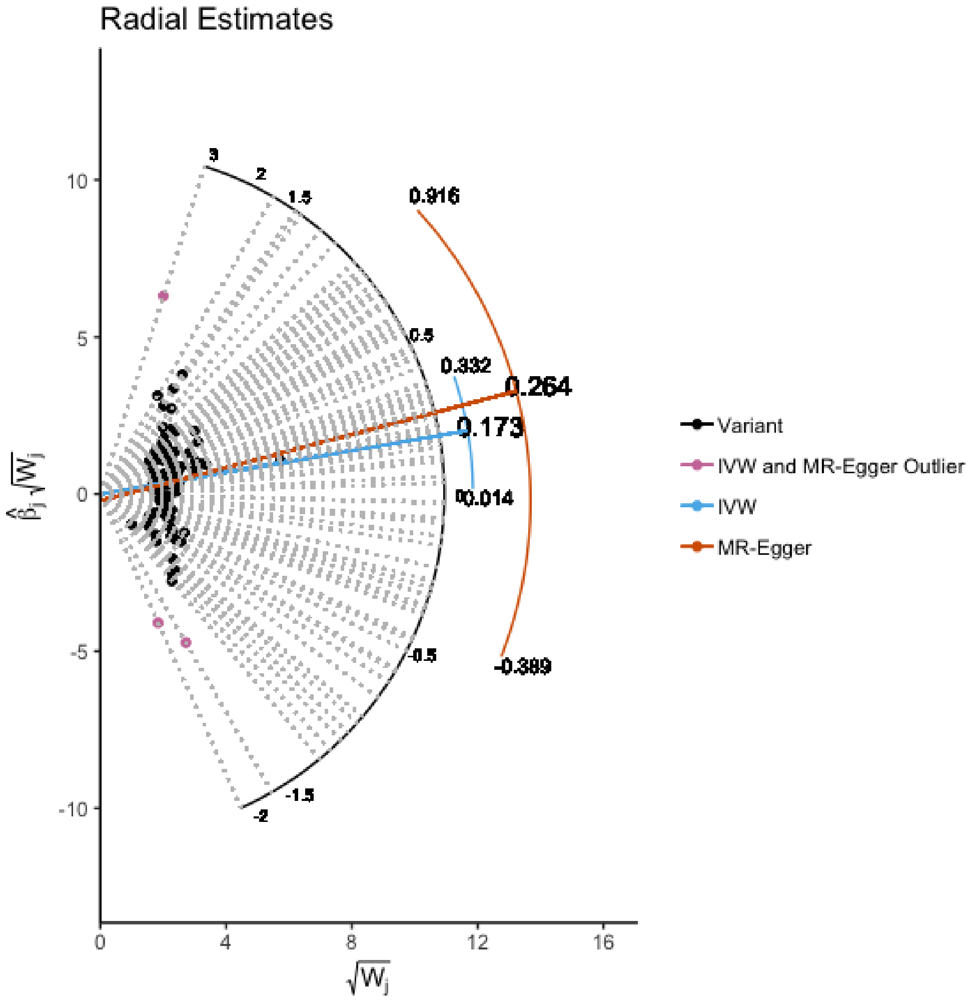

Radial curve displays the ratio estimate for each genetic variant, as well as the overall IVW (in blue) and MR Egger estimate (in orange). Data points with large contributions to Cochran's Q statistic are shown in pink, with an alpha of 0.05/82 = $6.1\text{x}10^{-4}$.

Supplementary Figure 6 - Radial MR plot for insomnia

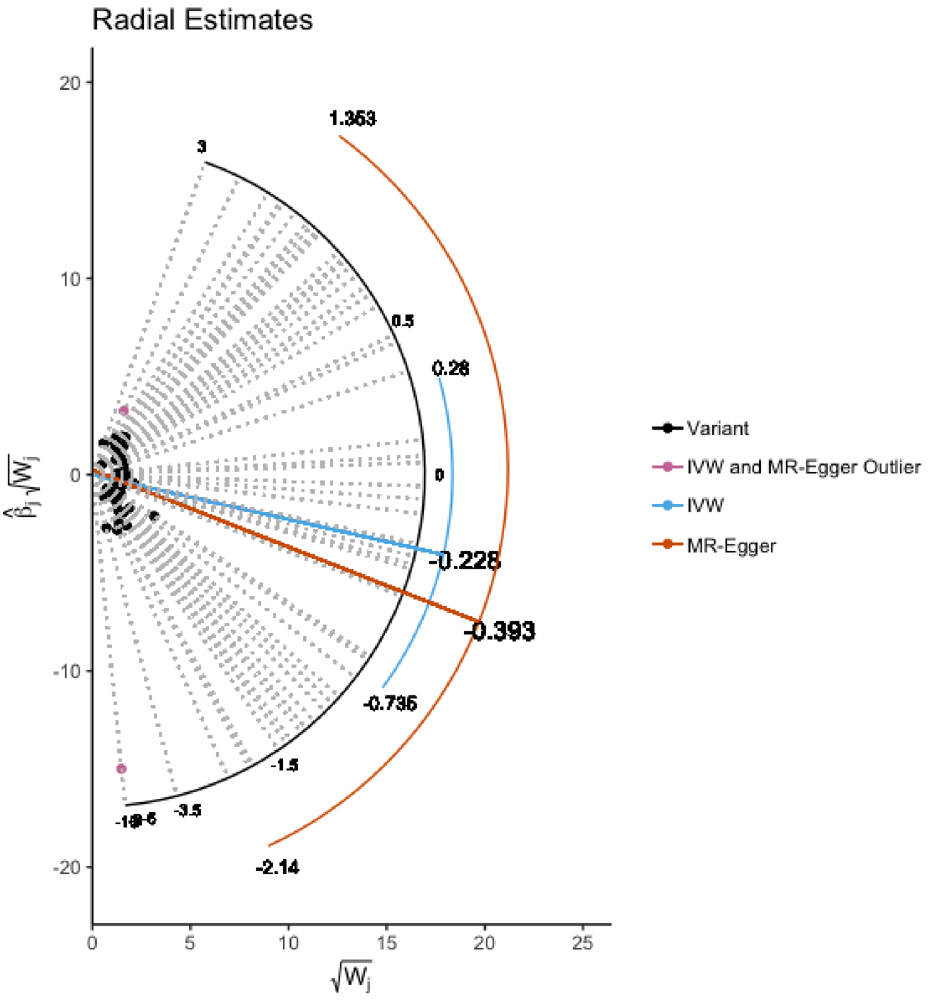

Radial curve displays the ratio estimate for each genetic variant, as well as the overall IVW (in blue) and MR Egger estimate (in orange). Data points with large contributions to Cochran's Q statistic are shown in pink, with an alpha of 0.05/50 = 1.0×10^{-3}.

Supplementary Figures 7a)-i) – Phenome-wide association study (PheWAS) outlier SNPs from Radial curves

Associations of SNPs with other traits in MR-Base (http://www.mrbase.org/) at $p<5x10^{-8}$ are annotated

rs1421085

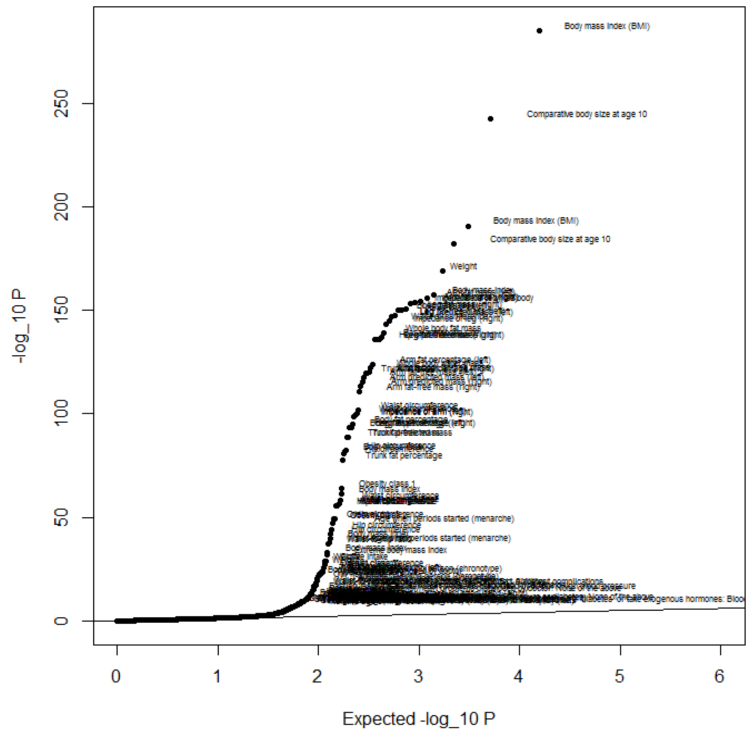

rs6718511

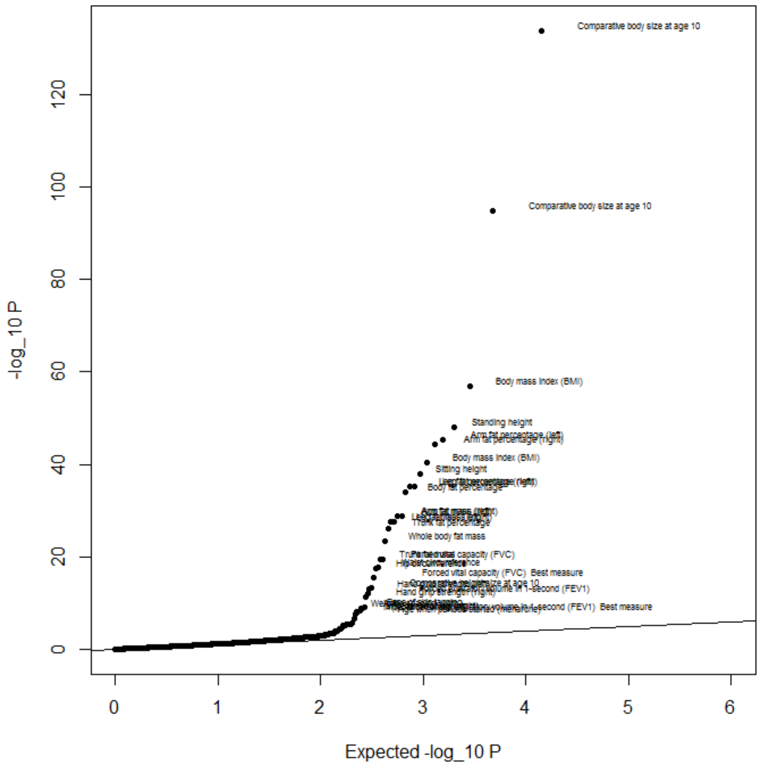

rs11681299

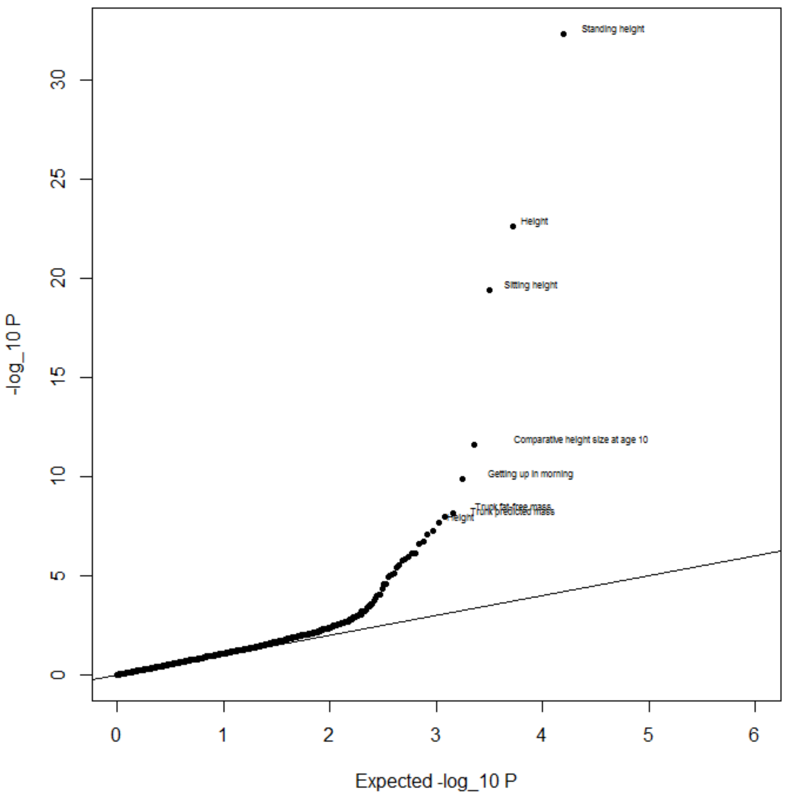

rs7626335

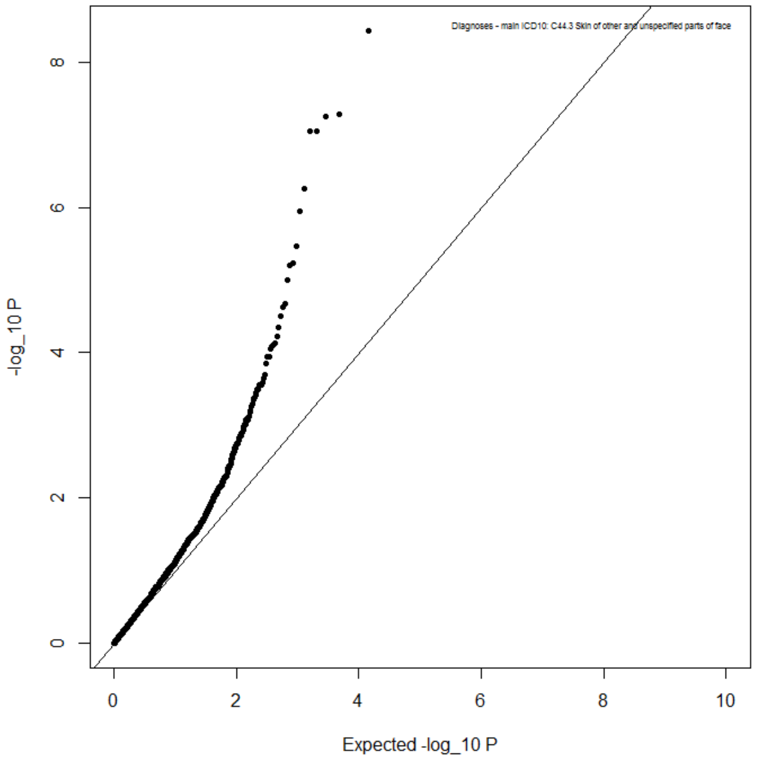

rs16939162

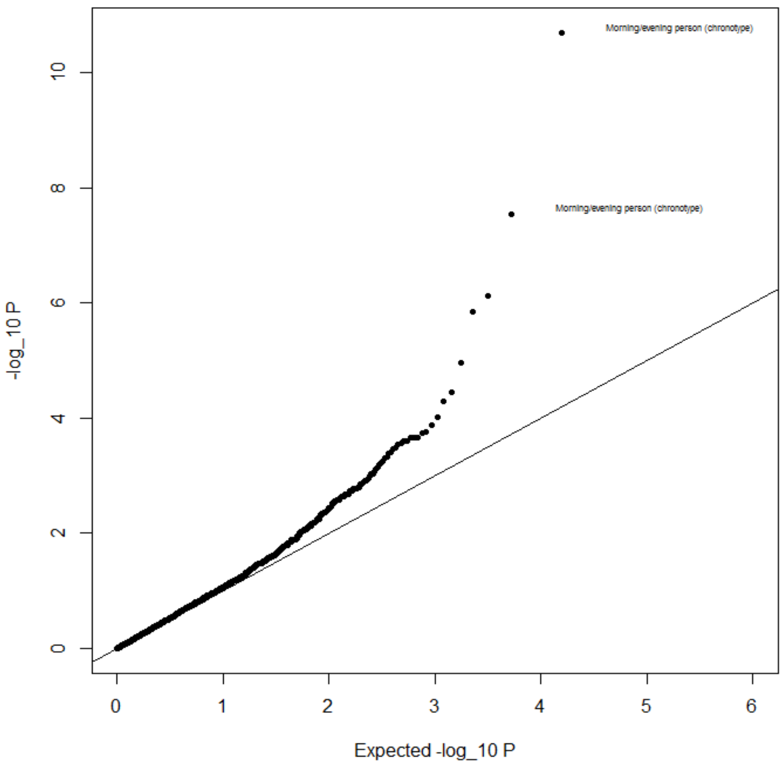

rs7225002

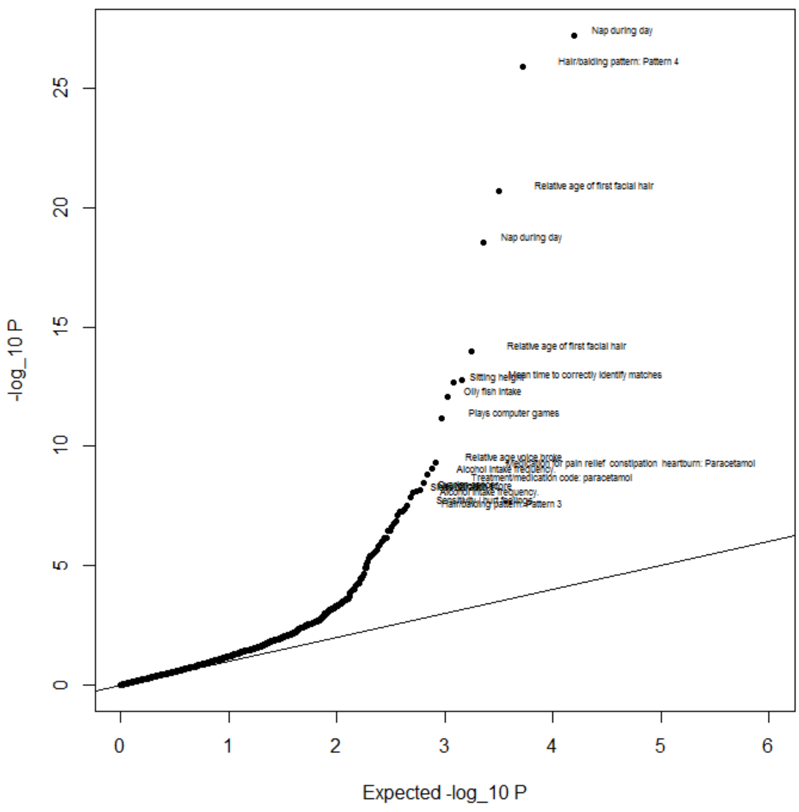

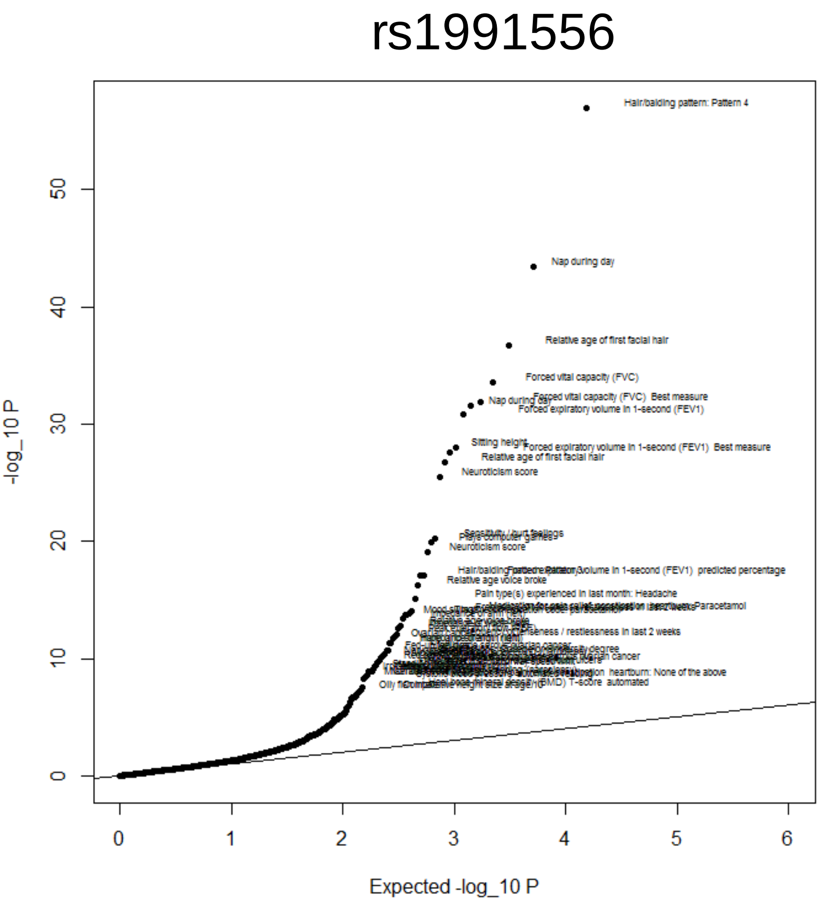
rs1991556
-log_10 P
Expected -log_10 P
0
10
20
30
40
50
0
1
2
3
4
5
6
Hair/balding pattern: Pattern 4
Nap during day
Relative age of first facial hair
Forced vital capacity (FVC)
Forced vital capacity (FVC), Best measure
Forced expiratory volume in 1-second (FEV1)
Sitting height
Forced expiratory volume in 1-second (FEV1), Best measure
Relative age of first facial hair
Neuroticism score
Plays computer games
Neuroticism score
Relative age voice broke
Pain type(s) experienced in last month: Headache

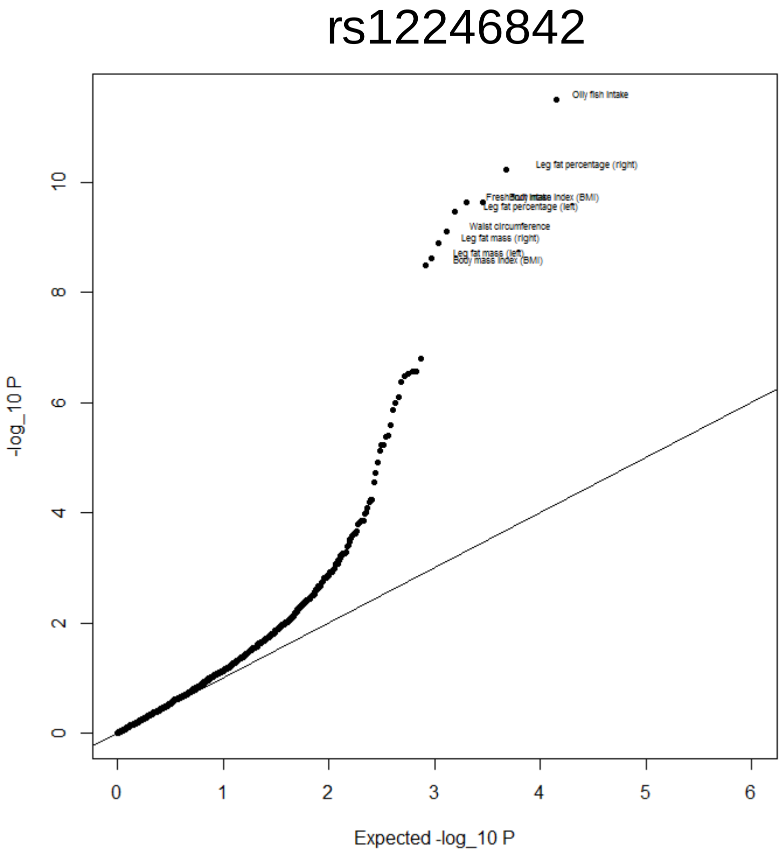
rs12246842
-log_10 P
Expected -log_10 P
0
2
4
6
8
10
0
1
2
3
4
5
6
Oily fish intake
Leg fat percentage (right)
Body mass index (BMI)
Leg fat percentage (left)
Waist circumference
Leg fat mass (right)
Leg fat mass (left)
Body mass index (BMI)

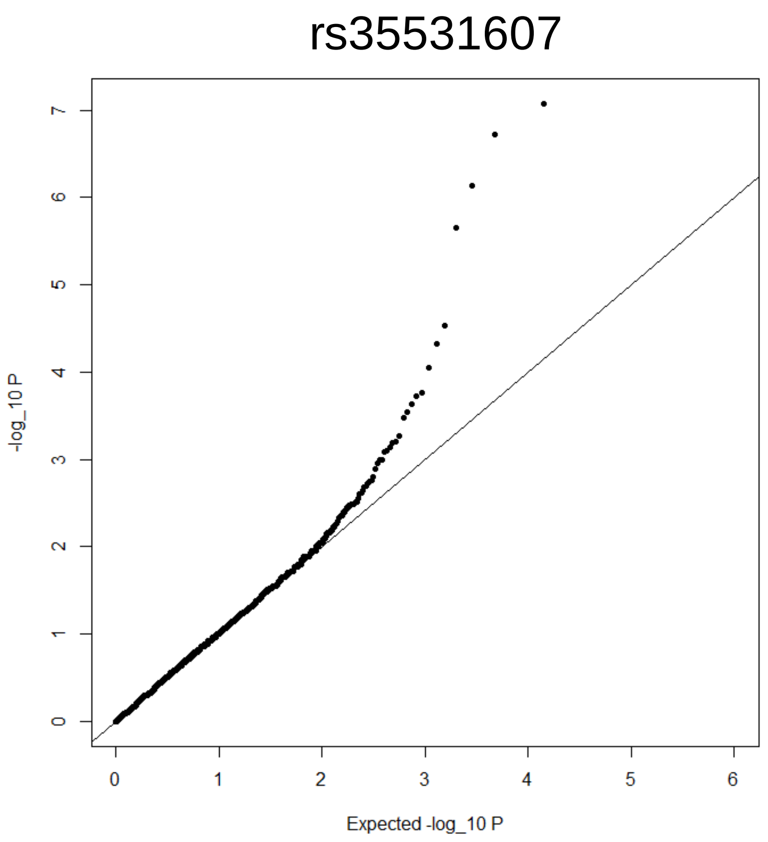

Supplementary Figure 8 - Scatter plot of individual SNP-L5 timing and SNP-breast cancer associations with overlay of causal estimate from each MR test in two-sample MR analysis

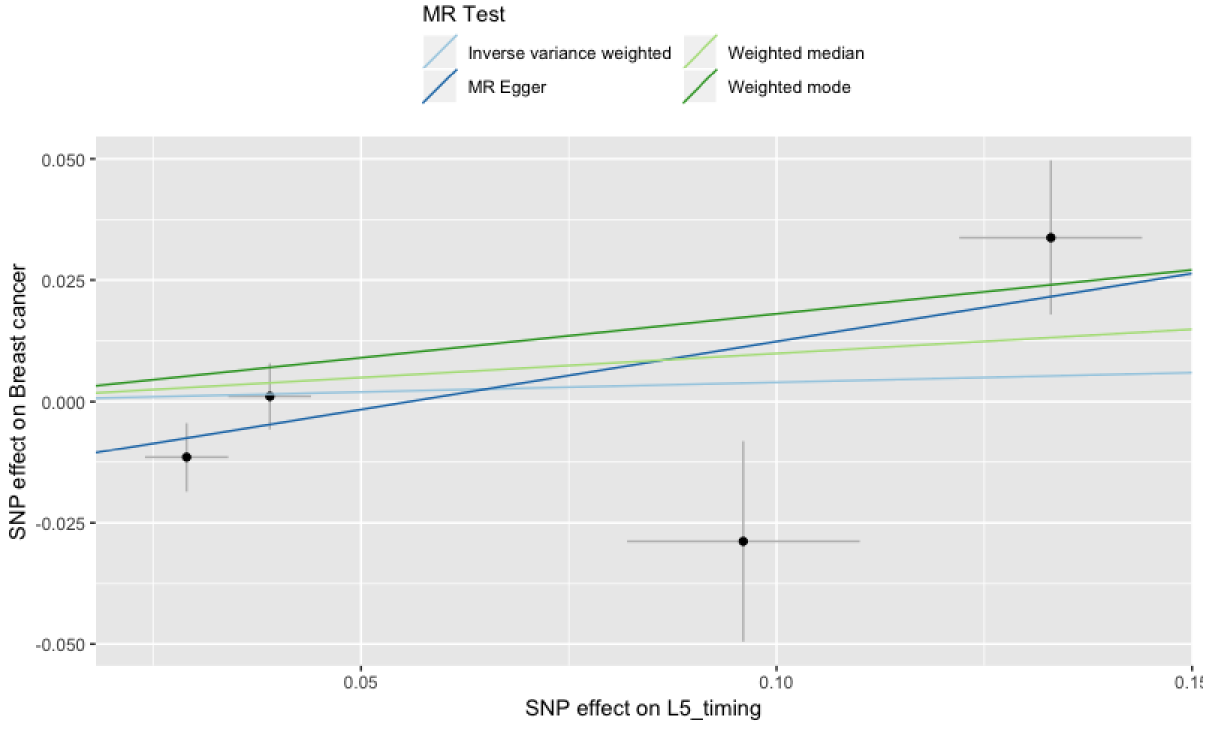

Supplementary Figure 9 - Scatter plot of individual SNP-nocturnal sleep duration and SNP-breast cancer associations with overlay of causal estimate from each MR test in two-sample MR analysis

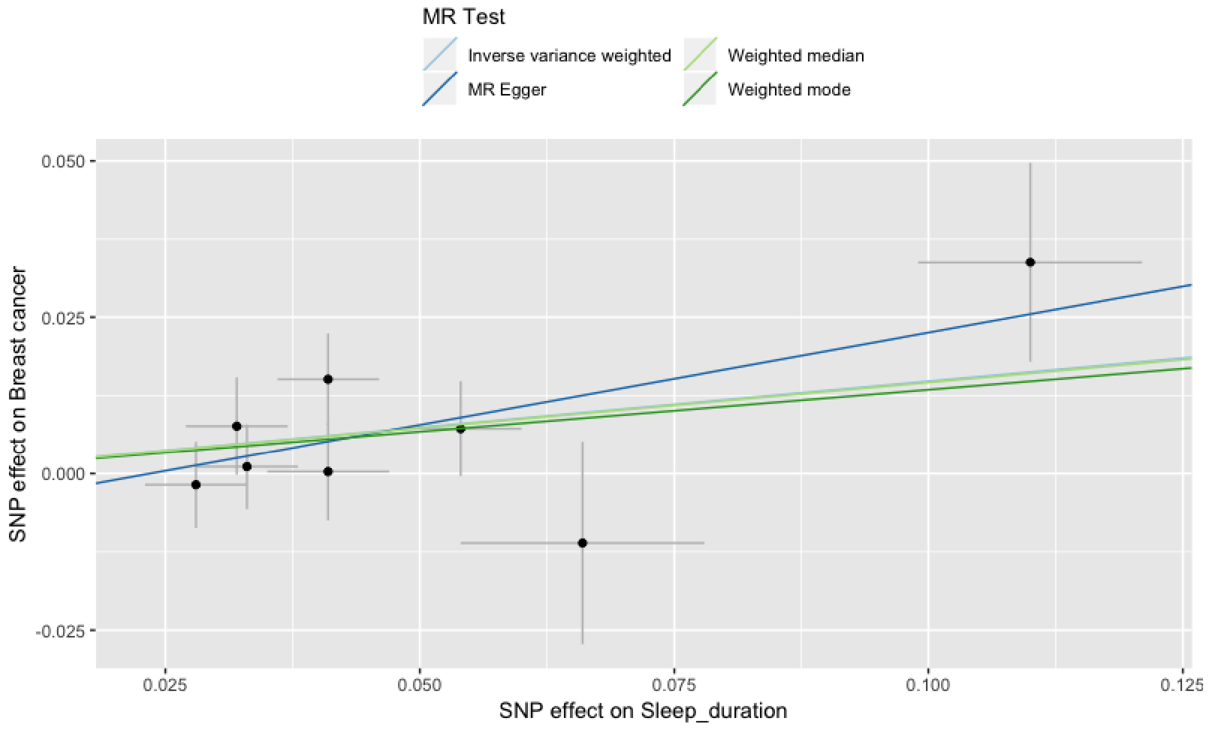

Supplementary Figure 10 - Scatter plot of individual SNP-nocturnal sleep episodes and SNP-breast cancer associations with overlay of causal estimate from each MR test in two-sample MR analysis

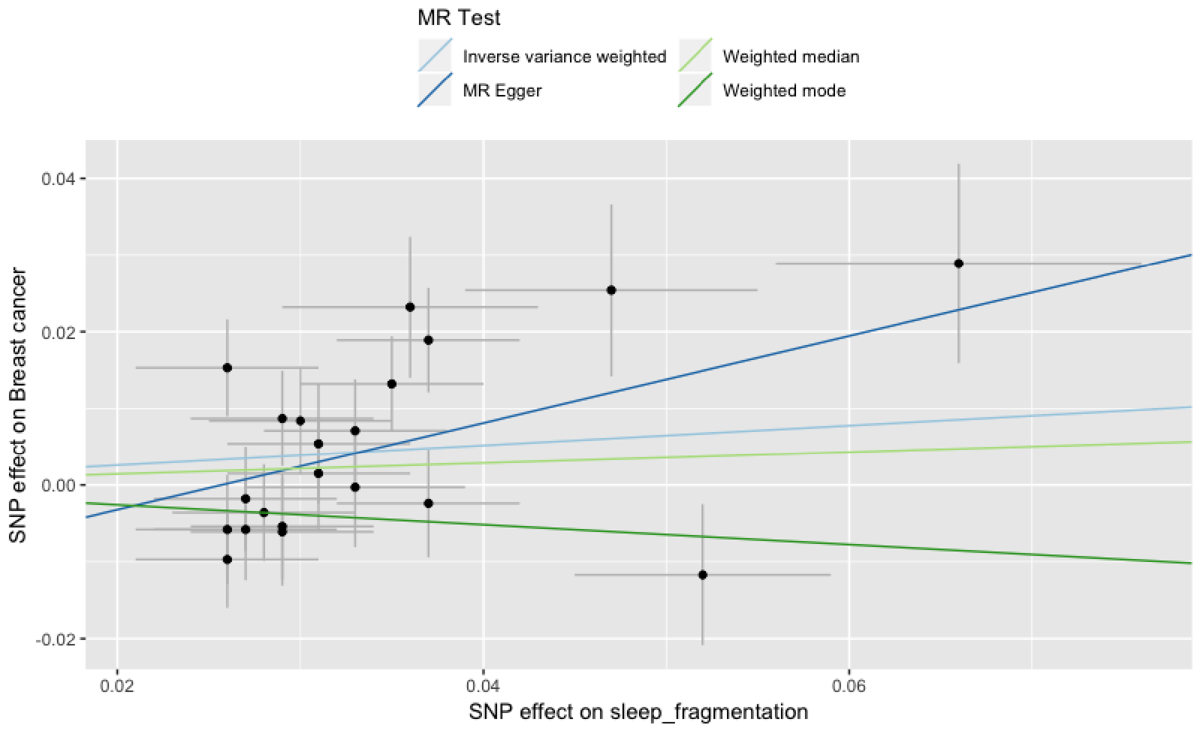

Supplementary Table 1 - SNP list and female-specific effect estimates of chronotype SNPs identified in UKBiobank [Jones et al, 2019]

SNP	effect_allele	other_allele	eaf	beta	se	pval	N	Bonferroni replication in 23andMe
rs10058356	C	T	0.301253	0.015206	0.004025	1.80E-04	244207	N
rs10109566	A	G	0.491754	-0.0182	0.0037	2.00E-06	244207	N
rs1013987	T	C	0.404791	-0.01703	0.003768	4.70E-06	244207	N
rs10175975	C	T	0.819386	-0.0243	0.004816	4.20E-07	244207	N
rs10237162	T	C	0.72589	0.015941	0.004133	1.00E-04	244207	Y
rs10254050	C	G	0.186747	-0.02287	0.004725	1.90E-06	244207	Y
rs1025601	C	T	0.614594	0.013193	0.003841	6.00E-04	244207	N
rs10402849	C	T	0.798421	-0.02413	0.004606	1.30E-07	244207	N
rs10520176	T	C	0.502199	0.022265	0.003685	9.20E-10	244207	Y
rs1061032	T	G	0.0886	0.043095	0.006538	4.40E-11	244207	Y
rs1064213	G	A	0.521392	-0.02134	0.003686	5.50E-09	244207	Y
rs10742179	A	G	0.259594	0.018248	0.004201	1.40E-05	244207	Y
rs10759208	T	C	0.608469	-0.01786	0.003792	3.60E-06	244207	Y
rs10762434	G	C	0.223327	-0.01676	0.004418	1.10E-04	244207	Y
rs10818834	T	C	0.733786	0.024967	0.004183	3.40E-09	244207	Y
rs10830107	A	G	0.797213	0.012411	0.004644	7.70E-03	244207	Y
rs10832648	C	A	0.804115	0.022294	0.004649	1.20E-06	244207	Y
rs10838687	T	G	0.790158	0.015533	0.004523	5.00E-04	244207	Y
rs10877962	C	T	0.586437	-0.01238	0.003757	1.40E-03	244207	Y
rs10916892	T	C	0.623513	-0.01816	0.003814	1.10E-06	244207	Y
rs10917513	C	T	0.344315	0.015409	0.003873	1.60E-04	244207	Y
rs10951325	T	C	0.630356	0.02672	0.003842	3.10E-12	244207	Y
rs10988239	C	T	0.487457	0.019559	0.003746	2.10E-07	244207	N
rs11032362	G	A	0.909313	-0.04022	0.006412	2.70E-10	244207	Y
rs11102807	A	G	0.537738	-0.0112	0.00371	1.60E-03	244207	N
rs111261826	A	C	0.679394	-0.01782	0.003945	7.80E-06	244207	Y
rs11152350	A	C	0.470195	-0.01622	0.003696	9.20E-06	244207	Y
rs11154718	T	C	0.42842	-0.01344	0.003731	2.70E-04	244207	Y
rs11165655	G	A	0.475433	0.016465	0.003691	7.50E-06	244207	Y
rs111867612	C	A	0.895349	0.022493	0.006067	1.40E-04	244207	N
rs11200159	C	A	0.345658	0.01655	0.003878	1.80E-05	244207	N

rs11208844	G	A	0.861859	0.029147	0.005335	5.50E-08	244207	N
rs113161209	G	A	0.929226	-0.02751	0.007279	1.40E-04	244207	N
rs113851554	G	T	0.942663	0.034637	0.008211	1.20E-05	244207	Y
rs1144566	T	C	0.029852	0.119435	0.010791	7.20E-29	244207	Y
rs114848860	A	T	0.97566	-0.05782	0.011987	2.00E-06	244207	Y
rs115073088	A	G	0.97666	-0.0582	0.012324	2.10E-06	244207	Y
rs11545787	G	A	0.749535	0.022108	0.004302	3.90E-07	244207	Y
rs11588913	G	A	0.601206	0.016312	0.003755	1.90E-05	244207	N
rs11597421	G	A	0.49896	0.011519	0.003753	2.60E-03	244207	Y
rs11611435	T	C	0.551226	0.015622	0.003705	2.20E-05	244207	N
rs1163238	G	A	0.605443	0.017097	0.003774	4.10E-06	244207	N
rs1163628	A	C	0.857442	-0.01678	0.005261	1.40E-03	244207	Y
rs11641239	C	T	0.711368	-0.01774	0.004065	2.50E-05	244207	N
rs11670534	C	T	0.834755	0.022168	0.004973	7.20E-06	244207	N
rs11677484	G	T	0.744581	-0.02151	0.004241	4.60E-07	244207	N
rs11678584	A	T	0.861363	-0.0186	0.005342	7.50E-04	244207	Y
rs11681299	C	T	0.712706	-0.01242	0.004062	1.80E-03	244207	Y
rs11788633	C	G	0.653058	0.011139	0.003873	4.40E-03	244207	Y
rs118047999	G	C	0.750615	-0.0142	0.004269	5.30E-04	244207	Y
rs11845599	A	G	0.635139	-0.01351	0.003865	5.30E-04	244207	Y
rs12040629	G	A	0.839294	-0.04335	0.005006	6.10E-18	244207	Y
rs12051	A	G	0.612069	-0.01244	0.003788	8.70E-04	244207	Y
rs12065331	C	T	0.690907	0.015149	0.004018	1.30E-04	244207	Y
rs12140153	G	T	0.905255	0.02302	0.00645	2.30E-04	244207	Y
rs12195792	T	A	0.731539	-0.01088	0.004154	7.40E-03	244207	Y
rs12206814	G	C	0.508127	-0.01666	0.003741	7.60E-06	244207	N
rs1221502	A	C	0.737691	0.019077	0.00418	4.20E-06	244207	N
rs12249410	G	T	0.889788	0.023028	0.005997	1.30E-04	244207	N
rs12298405	C	T	0.673325	0.011937	0.003928	2.90E-03	244207	N
rs12378543	C	T	0.613442	0.012555	0.003796	8.30E-04	244207	N
rs12380242	T	C	0.502176	-0.01529	0.003682	2.40E-05	244207	N
rs12436039	T	C	0.882267	0.012275	0.005786	3.10E-02	244207	Y
rs12442008	C	T	0.747261	-0.01402	0.004264	8.20E-04	244207	Y

rs12442674	A	C	0.72465	0.015557	0.004132	1.30E-04	244207	Y
rs12445235	G	C	0.590119	0.012143	0.003757	1.40E-03	244207	Y
rs12464387	A	G	0.460119	-0.01473	0.003696	7.00E-05	244207	Y
rs12470914	T	A	0.897414	-0.02168	0.006075	5.10E-04	244207	Y
rs12518401	G	A	0.612327	0.013171	0.003902	6.90E-04	244207	Y
rs12600452	G	A	0.794392	-0.01114	0.004569	1.30E-02	244207	Y
rs12631477	T	C	0.799958	0.017855	0.00461	7.20E-05	244207	N
rs12636669	C	T	0.918173	-0.03821	0.006821	4.10E-08	244207	Y
rs12771973	G	A	0.746781	0.013772	0.004239	1.10E-03	244207	Y
rs1278402	A	G	0.733057	0.008571	0.004175	3.40E-02	244207	Y
rs12808544	C	A	0.760024	0.016738	0.004317	1.00E-04	244207	Y
rs12871550	G	A	0.678061	-0.02145	0.003939	3.10E-08	244207	Y
rs12927162	A	G	0.721067	0.033635	0.004111	3.10E-16	244207	Y
rs12950382	A	G	0.72498	0.011553	0.004138	5.10E-03	244207	Y
rs12969848	C	T	0.470014	-0.02472	0.003708	6.20E-11	244207	Y
rs13004345	C	T	0.347258	0.010813	0.00386	5.10E-03	244207	Y
rs13011556	C	G	0.761834	-0.02603	0.004335	4.90E-09	244207	N
rs13065394	G	T	0.711843	0.01901	0.004061	4.00E-06	244207	N
rs13172141	A	T	0.566628	0.016328	0.003714	1.20E-05	244207	Y
rs13203140	C	T	0.358707	0.012681	0.003841	1.00E-03	244207	Y
rs13377754	T	C	0.612366	0.030794	0.003781	4.60E-16	244207	Y
rs13414393	T	C	0.540593	-0.01502	0.0037	3.30E-05	244207	N
rs1398346	C	T	0.129344	-0.01785	0.005506	1.50E-03	244207	Y
rs139911	C	T	0.423256	0.026672	0.003744	1.80E-12	244207	Y
rs1421085	T	C	0.598789	-0.03013	0.003762	2.10E-15	244207	Y
rs1449403	G	A	0.87642	-0.01905	0.005587	3.00E-04	244207	Y
rs1468945	G	A	0.213997	0.020005	0.004488	5.40E-06	244207	Y
rs1474754	A	G	0.268116	-0.01041	0.004172	1.40E-02	244207	Y
rs149611468	T	C	0.988095	0.109072	0.017292	4.00E-10	244207	Y
rs1502249	A	G	0.524143	0.011267	0.003697	1.90E-03	244207	N
rs1508608	A	G	0.321234	0.012418	0.003946	2.60E-03	244207	Y
rs1559253	G	A	0.642895	-0.01187	0.003873	3.20E-03	244207	Y
rs1599374	G	A	0.487575	-0.016	0.003709	1.90E-05	244207	Y

rs16939162	A	G	0.830529	0.022552	0.004912	5.00E-06	244207	Y
rs17007397	C	G	0.576307	0.010728	0.003731	3.70E-03	244207	Y
rs17140201	G	A	0.825947	0.024083	0.004971	1.60E-06	244207	N
rs17302081	T	C	0.441674	0.017109	0.003705	5.40E-06	244207	N
rs1737893	C	T	0.618445	0.014053	0.003821	4.30E-04	244207	Y
rs17396357	C	T	0.620211	-0.00869	0.003799	1.60E-02	244207	Y
rs17448682	C	T	0.768059	-0.02208	0.004364	2.30E-07	244207	Y
rs17455138	T	C	0.765478	0.012273	0.004375	5.10E-03	244207	Y
rs17575798	G	A	0.806042	0.023425	0.004642	3.20E-07	244207	Y
rs17577073	A	C	0.565978	0.013068	0.003747	4.10E-04	244207	Y
rs17604349	G	A	0.820038	0.025667	0.004806	1.70E-07	244207	Y
rs17712705	A	G	0.330109	-0.00629	0.00392	1.10E-01	244207	Y
rs1788784	A	G	0.342826	-0.01054	0.003888	5.20E-03	244207	Y
rs1799464	A	G	0.289703	-0.01517	0.00407	2.20E-04	244207	N
rs1800828	C	G	0.746422	0.018242	0.004233	2.30E-05	244207	N
rs1811899	T	C	0.78979	-0.0105	0.004548	2.50E-02	244207	Y
rs184033703	G	A	0.942218	-0.03186	0.007914	4.20E-05	244207	Y
rs1843888	G	A	0.450213	-0.02778	0.003711	9.50E-14	244207	Y
rs187028	A	T	0.316767	-0.01515	0.003971	1.70E-04	244207	Y
rs1871729	A	G	0.682986	-0.01436	0.003948	1.90E-04	244207	Y
rs1873958	G	A	0.593468	-0.01491	0.003779	5.80E-05	244207	Y
rs1886205	C	A	0.239878	-0.01723	0.004333	4.50E-05	244207	Y
rs1931814	A	G	0.477702	0.013508	0.003684	2.40E-04	244207	Y
rs2011528	C	T	0.169189	0.0256	0.004918	1.20E-07	244207	Y
rs2050185	G	A	0.372649	-0.01463	0.003828	1.30E-04	244207	N
rs2072413	C	T	0.728238	0.014848	0.004163	5.70E-04	244207	N
rs2072727	T	C	0.436337	0.01564	0.003723	3.00E-05	244207	Y
rs2166559	T	C	0.860039	-0.02388	0.005308	3.50E-06	244207	N
rs2298117	T	C	0.45136	-0.01443	0.003703	1.20E-04	244207	N
rs2304467	C	G	0.600769	-0.01192	0.003787	1.10E-03	244207	N
rs2322605	G	A	0.519205	0.015991	0.00369	1.30E-05	244207	Y
rs2362775	T	C	0.526931	-0.01522	0.003706	6.10E-05	244207	N
rs2396004	A	G	0.432815	0.017773	0.003711	1.60E-06	244207	Y

rs2396719	G	A	0.752566	-0.01613	0.004285	1.60E-04	244207	Y
rs2433634	A	C	0.718103	-0.01218	0.004092	3.20E-03	244207	Y
rs247929	G	C	0.495008	-0.0218	0.00369	2.90E-09	244207	Y
rs2506089	T	G	0.569337	0.012764	0.00371	6.30E-04	244207	Y
rs2514214	A	G	0.268376	0.018453	0.004162	8.40E-06	244207	Y
rs2550298	C	T	0.623087	0.01982	0.003806	2.60E-07	244207	Y
rs2580160	A	G	0.55286	0.020163	0.003747	7.80E-08	244207	Y
rs2593487	G	A	0.659687	0.016256	0.0039	4.40E-05	244207	Y
rs2648721	T	G	0.701586	-0.01599	0.004025	7.80E-05	244207	N
rs2653349	A	G	0.215119	0.041211	0.004484	4.30E-20	244207	Y
rs2706762	C	T	0.85007	0.024221	0.00515	1.90E-06	244207	Y
rs2737245	G	T	0.719126	-0.02146	0.004115	3.10E-07	244207	Y
rs28380327	A	T	0.628707	0.01944	0.003806	4.90E-07	244207	Y
rs2844016	T	C	0.298643	0.014232	0.004036	4.80E-04	244207	Y
rs28458909	C	T	0.875737	0.051325	0.005579	1.80E-20	244207	Y
rs28459838	T	C	0.233788	0.01509	0.004381	6.30E-04	244207	Y
rs2850979	C	T	0.23949	0.018078	0.004327	2.50E-05	244207	N
rs2878172	A	G	0.566124	-0.01267	0.003743	1.10E-03	244207	Y
rs2881955	C	T	0.72137	-0.01704	0.004113	3.20E-05	244207	Y
rs2901796	A	G	0.396085	0.013284	0.003776	3.20E-04	244207	Y
rs2916148	G	A	0.544243	-0.01057	0.003751	4.00E-03	244207	Y
rs2944831	G	A	0.701247	-0.01529	0.004048	2.40E-04	244207	N
rs295268	T	C	0.743865	-0.02227	0.004226	8.10E-08	244207	Y
rs2978382	T	C	0.589997	0.017088	0.003767	7.70E-06	244207	N
rs2979139	A	G	0.503737	-0.01687	0.003691	5.60E-06	244207	Y
rs301218	G	A	0.609239	0.014277	0.003763	1.50E-04	244207	Y
rs308521	T	C	0.60266	0.020796	0.003779	2.30E-08	244207	Y
rs3100052	A	G	0.386774	0.014204	0.003783	2.00E-04	244207	N
rs3138490	T	A	0.483974	-0.00874	0.003717	2.40E-02	244207	Y
rs34054660	A	G	0.573234	0.010992	0.003727	3.60E-03	244207	Y
rs34329963	C	T	0.886403	0.027949	0.005823	1.10E-06	244207	Y
rs34509802	G	A	0.82104	-0.01463	0.004793	1.80E-03	244207	Y
rs34967119	G	A	0.499188	-0.0124	0.003682	8.40E-04	244207	Y

rs35346733	G	A	0.80578	0.018046	0.004682	1.10E-04	244207	Y
rs35524253	G	A	0.64302	-0.01421	0.003854	1.40E-04	244207	Y
rs359248	T	G	0.448197	-0.01828	0.003716	1.30E-06	244207	Y
rs36055559	G	A	0.826377	0.026217	0.005093	2.40E-07	244207	Y
rs3743794	G	A	0.394448	0.015912	0.003787	1.60E-05	244207	N
rs3760381	G	A	0.74908	-0.01486	0.00427	4.30E-04	244207	Y
rs3782860	C	T	0.451453	-0.01577	0.003708	2.50E-05	244207	Y
rs3796618	T	A	0.469141	0.013472	0.003694	2.10E-04	244207	N
rs3807651	A	T	0.491394	0.016943	0.0037	7.40E-06	244207	N
rs3808964	G	T	0.365287	-0.00989	0.003834	1.20E-02	244207	N
rs3815983	C	T	0.640034	0.015764	0.003877	4.40E-05	244207	Y
rs3850174	T	A	0.743077	0.018519	0.004251	1.20E-05	244207	Y
rs3857599	C	A	0.836856	-0.02158	0.004985	2.60E-05	244207	N
rs3867239	G	A	0.621303	-0.0136	0.003811	3.30E-04	244207	Y
rs3923809	A	G	0.697144	-0.01222	0.004004	2.90E-03	244207	Y
rs3955311	C	T	0.849507	-0.02025	0.00517	8.60E-05	244207	N
rs4027217	C	A	0.788823	0.017344	0.004503	8.20E-05	244207	N
rs412000	G	C	0.443291	0.019194	0.003713	2.10E-07	244207	Y
rs4121878	G	C	0.492475	-0.01347	0.003686	2.10E-04	244207	Y
rs42210	G	C	0.288223	0.023294	0.004089	1.20E-08	244207	Y
rs4236237	C	A	0.402427	0.010424	0.003758	5.20E-03	244207	Y
rs4241964	T	G	0.52441	-0.02108	0.003708	1.70E-08	244207	N
rs4269995	C	T	0.747922	0.022489	0.004239	1.60E-07	244207	Y
rs4365329	A	T	0.541802	-0.01514	0.00371	4.90E-05	244207	Y
rs4419127	A	G	0.66419	0.023328	0.003914	1.40E-09	244207	Y
rs4535583	C	T	0.303628	-0.01815	0.004001	1.00E-05	244207	Y
rs4550384	T	G	0.753947	0.014109	0.004289	1.10E-03	244207	Y
rs4550782	G	T	0.331489	-0.0179	0.003911	4.50E-06	244207	Y
rs45597035	A	G	0.653408	-0.01265	0.0039	1.00E-03	244207	N
rs465670	C	T	0.457114	-0.01189	0.003696	1.80E-03	244207	Y
rs4657983	G	A	0.346577	0.012769	0.00387	9.30E-04	244207	Y
rs4666682	G	A	0.823839	0.020085	0.004821	2.80E-05	244207	N
rs4672458	C	T	0.526608	0.012127	0.003681	1.10E-03	244207	N

rs4690085	A	G	0.529326	-0.02067	0.003687	3.60E-08	244207	N
rs4698678	C	G	0.281864	0.010867	0.004095	8.20E-03	244207	Y
rs4729854	T	A	0.517257	0.030265	0.003771	7.40E-16	244207	Y
rs4761989	C	T	0.130743	0.01798	0.00547	1.20E-03	244207	Y
rs4775086	G	A	0.761806	0.015789	0.004337	2.30E-04	244207	Y
rs4785296	G	C	0.769039	-0.0134	0.004369	2.50E-03	244207	Y
rs4800998	T	A	0.81421	-0.01669	0.004752	4.80E-04	244207	Y
rs4804951	A	G	0.330179	0.017697	0.003942	7.40E-06	244207	N
rs481214	A	T	0.602084	0.012547	0.003763	5.10E-04	244207	N
rs4860734	G	A	0.717062	-0.00959	0.004132	2.10E-02	244207	N
rs486416	G	A	0.36641	0.020546	0.003808	8.50E-08	244207	N
rs487722	G	T	0.787814	-0.01634	0.004513	2.70E-04	244207	N
rs4878734	A	T	0.518903	0.01716	0.003685	3.90E-06	244207	N
rs4903203	A	G	0.32129	0.012944	0.003951	8.80E-04	244207	Y
rs4923541	C	T	0.490126	-0.01523	0.003684	6.50E-05	244207	Y
rs4936290	A	C	0.649766	-0.02187	0.003881	3.20E-08	244207	N
rs495593	G	A	0.257062	-0.018	0.004223	2.30E-05	244207	Y
rs497338	C	T	0.718638	-0.01036	0.004098	1.30E-02	244207	Y
rs5016898	C	T	0.573872	0.011033	0.003743	2.70E-03	244207	Y
rs555784	T	A	0.616195	0.013819	0.003796	2.20E-04	244207	Y
rs55846845	G	A	0.477815	0.016734	0.003694	5.00E-06	244207	N
rs56113850	T	C	0.421286	-0.00954	0.003734	8.00E-03	244207	Y
rs57236847	C	G	0.603056	0.011981	0.003773	2.00E-03	244207	Y
rs58681483	A	G	0.922608	0.015478	0.006901	2.30E-02	244207	Y
rs58876439	G	A	0.930588	-0.02103	0.007261	3.20E-03	244207	Y
rs59986227	C	G	0.742849	-0.01995	0.004258	2.20E-06	244207	Y
rs6007594	G	A	0.741422	0.022147	0.004196	1.50E-07	244207	Y
rs6047481	A	T	0.672205	0.010237	0.003941	1.10E-02	244207	Y
rs60616179	A	G	0.944749	0.032556	0.008123	4.30E-05	244207	N
rs6131805	T	G	0.399343	0.017024	0.00384	7.90E-06	244207	Y
rs6131942	A	G	0.419283	-0.02291	0.00375	1.30E-09	244207	Y
rs61773390	G	T	0.803965	-0.04051	0.004631	3.00E-18	244207	Y
rs61875203	C	T	0.722947	-0.01699	0.004118	5.50E-05	244207	Y

rs61990287	C	A	0.717135	-0.01042	0.004106	9.60E-03	244207	Y
rs62082402	G	T	0.809041	-0.02792	0.004698	1.40E-09	244207	Y
rs62124718	A	G	0.894152	-0.02272	0.005987	1.20E-04	244207	Y
rs621421	T	C	0.620807	-0.02188	0.003803	7.60E-09	244207	Y
rs62182135	C	A	0.667482	0.016191	0.003906	4.60E-05	244207	Y
rs62465218	C	A	0.851561	0.017501	0.005186	7.30E-04	244207	Y
rs62479736	T	G	0.293484	0.013954	0.004047	4.40E-04	244207	Y
rs62553781	C	T	0.965198	0.054665	0.010077	6.80E-08	244207	N
rs6429233	G	A	0.547121	-0.01106	0.003707	2.80E-03	244207	N
rs6433478	T	C	0.455374	-0.01508	0.003717	3.80E-05	244207	Y
rs6440833	G	A	0.537362	-0.01227	0.003707	1.10E-03	244207	Y
rs6468316	C	T	0.524992	0.015476	0.003689	2.60E-05	244207	N
rs6477309	C	T	0.334138	-0.01674	0.003926	2.00E-05	244207	Y
rs6544906	A	C	0.565169	0.015475	0.003714	3.30E-05	244207	Y
rs6560218	C	T	0.483134	0.016306	0.003725	1.60E-05	244207	N
rs6573308	C	T	0.613104	-0.01334	0.003804	4.70E-04	244207	Y
rs662094	G	A	0.505458	-0.0123	0.003686	1.10E-03	244207	Y
rs66507804	T	C	0.794545	-0.01664	0.004561	2.50E-04	244207	Y
rs66617308	T	C	0.670722	0.012368	0.003922	1.20E-03	244207	N
rs6665637	G	A	0.718849	0.013028	0.004182	1.20E-03	244207	Y
rs6690292	C	T	0.271811	0.010684	0.004131	8.40E-03	244207	N
rs6718511	A	G	0.547724	0.01059	0.003698	4.90E-03	244207	Y
rs6727752	G	A	0.626643	-0.01834	0.003801	2.10E-06	244207	N
rs6794796	A	G	0.286835	0.015376	0.004066	1.70E-04	244207	N
rs67988891	C	G	0.681164	-0.02267	0.003958	8.60E-09	244207	Y
rs6816922	C	A	0.464183	0.013592	0.003706	1.90E-04	244207	N
rs6838677	A	C	0.669349	-0.01252	0.003917	1.50E-03	244207	N
rs6846730	C	T	0.767387	0.021376	0.004354	4.60E-07	244207	Y
rs695459	C	T	0.610683	0.014975	0.003786	6.60E-05	244207	N
rs6958557	T	G	0.606245	0.010943	0.003765	4.20E-03	244207	Y
rs6967481	C	T	0.503323	-0.01636	0.003697	4.20E-06	244207	Y
rs6968240	C	A	0.5715	-0.00809	0.003744	3.60E-02	244207	Y
rs6988733	C	T	0.652862	-0.01344	0.003871	5.40E-04	244207	Y

rs6993892	T	C	0.608027	-0.02509	0.003778	3.90E-11	244207	Y
rs7006885	G	A	0.711496	-0.01577	0.004074	1.40E-04	244207	Y
rs710284	T	C	0.584478	0.014448	0.003744	1.40E-04	244207	Y
rs711098	A	C	0.402819	0.012088	0.003769	1.20E-03	244207	Y
rs7111582	G	A	0.104195	0.029951	0.006073	9.80E-07	244207	Y
rs7143933	T	G	0.263371	0.008492	0.004186	3.90E-02	244207	Y
rs71523448	G	C	0.921131	0.028505	0.006944	5.00E-05	244207	Y
rs7203707	C	A	0.483331	0.017318	0.0037	2.70E-06	244207	N
rs7225002	A	G	0.58435	-0.01145	0.003746	3.10E-03	244207	N
rs7248205	C	T	0.398392	-0.01684	0.003778	4.50E-06	244207	Y
rs72720396	A	G	0.770306	-0.02722	0.004373	5.50E-10	244207	Y
rs72729847	T	C	0.803615	-0.01529	0.004638	8.80E-04	244207	Y
rs72773411	G	A	0.839835	-0.01764	0.005045	3.60E-04	244207	Y
rs72790386	G	T	0.966757	-0.03149	0.010278	1.40E-03	244207	Y
rs72796401	T	A	0.811273	-0.02415	0.004703	1.20E-07	244207	Y
rs72829706	A	G	0.9604	0.05274	0.009486	2.60E-08	244207	N
rs72841368	A	T	0.812041	-0.01739	0.00472	3.30E-04	244207	Y
rs72950188	T	C	0.924267	0.02114	0.006957	2.10E-03	244207	Y
rs72966564	C	T	0.751019	0.017798	0.004288	4.50E-05	244207	N
rs7298532	T	C	0.717599	0.017273	0.004088	2.30E-05	244207	N
rs7299922	A	G	0.629841	0.016088	0.003837	2.40E-05	244207	N
rs73026775	G	A	0.871857	0.02482	0.005717	1.30E-05	244207	Y
rs7304278	A	G	0.275159	-0.02179	0.004143	2.30E-07	244207	Y
rs73050286	T	C	0.783806	0.019599	0.004471	1.10E-05	244207	Y
rs7429614	G	T	0.582467	-0.01764	0.003733	1.80E-06	244207	Y
rs74357745	A	G	0.87892	0.027574	0.005652	1.40E-06	244207	N
rs747003	T	C	0.606864	0.015846	0.003778	3.70E-05	244207	N
rs75120545	C	T	0.969849	-0.04998	0.011366	1.10E-05	244207	Y
rs7602499	C	T	0.654649	-0.0107	0.003887	9.50E-03	244207	Y
rs76064513	C	T	0.871324	-0.01416	0.005566	1.00E-02	244207	Y
rs7626335	A	C	0.329545	-0.01158	0.003931	2.60E-03	244207	Y
rs7649164	T	G	0.580169	0.01279	0.003806	9.80E-04	244207	Y
rs76518095	C	T	0.922418	-0.02172	0.006951	1.90E-03	244207	Y

rs766406	G	T	0.366012	0.01107	0.00382	3.60E-03	244207	Y
rs7700110	G	A	0.741351	-0.01148	0.004205	4.60E-03	244207	Y
rs7701529	A	T	0.237837	-0.01442	0.004355	8.80E-04	244207	Y
rs7721608	G	T	0.535478	-0.01174	0.003693	1.70E-03	244207	Y
rs77248969	G	A	0.910024	0.022816	0.006434	3.20E-04	244207	Y
rs7735794	G	A	0.776243	-0.02301	0.00467	9.50E-07	244207	N
rs77960	G	A	0.67207	-0.01805	0.003929	3.70E-06	244207	N
rs7845620	A	C	0.834937	-0.02093	0.004964	2.80E-05	244207	Y
rs78580841	C	T	0.928526	-0.03656	0.007338	5.90E-07	244207	N
rs7900191	C	T	0.604066	0.015211	0.003781	6.60E-05	244207	N
rs7943634	C	T	0.691234	0.015607	0.003992	6.50E-05	244207	N
rs7959983	T	C	0.596475	-0.02002	0.003745	6.30E-08	244207	Y
rs7975791	C	T	0.960531	-0.03805	0.009447	2.60E-05	244207	N
rs80097534	G	T	0.901085	0.025784	0.006232	4.80E-05	244207	N
rs80271258	C	T	0.912705	0.064153	0.006529	9.80E-23	244207	Y
rs8044054	C	T	0.611025	-0.02179	0.003781	1.30E-08	244207	Y
rs8072058	T	A	0.218403	0.01687	0.004465	1.40E-04	244207	Y
rs812925	C	G	0.649689	-0.02086	0.003852	5.40E-08	244207	Y
rs848552	C	G	0.472651	-0.01691	0.003677	4.90E-06	244207	Y
rs909757	T	C	0.631043	0.009949	0.00381	7.60E-03	244207	N
rs9347926	A	T	0.445971	0.016547	0.003696	7.20E-06	244207	Y
rs9348050	T	C	0.488745	0.018051	0.003693	4.80E-07	244207	N
rs9381812	A	G	0.703834	-0.02258	0.004039	2.30E-08	244207	Y
rs938836	G	A	0.534187	0.011848	0.003696	1.50E-03	244207	Y
rs9394154	C	G	0.430189	-0.01847	0.003722	5.30E-07	244207	N
rs9416744	A	C	0.256261	0.022289	0.004224	1.70E-07	244207	Y
rs9436119	G	A	0.60535	-0.02407	0.003756	1.50E-10	244207	Y
rs9465253	C	T	0.721148	-0.01877	0.004121	3.20E-06	244207	Y
rs9476310	C	T	0.48956	-0.01235	0.00369	8.60E-04	244207	Y
rs9479402	T	C	0.989581	-0.1356	0.018114	2.90E-14	244207	Y
rs9496623	A	G	0.738586	-0.01471	0.004332	8.40E-04	244207	N
rs9558942	T	C	0.673515	-0.00924	0.003927	1.90E-02	244207	Y
rs9571526	T	G	0.769707	-0.01583	0.004378	1.90E-04	244207	Y

rs9573980	A	G	0.966247	0.086736	0.010212	3.70E-17	244207	Y
rs9597241	A	C	0.811214	0.028277	0.00474	2.20E-09	244207	Y
rs9611597	A	T	0.838392	0.02517	0.005054	6.80E-07	244207	Y
rs962961	C	T	0.671957	0.018243	0.003932	4.30E-06	244207	Y
rs9636202	G	A	0.733139	0.021344	0.004187	2.80E-07	244207	N
rs9664044	C	T	0.767246	0.015076	0.004363	5.70E-04	244207	N
rs975025	C	T	0.922515	0.038646	0.006868	1.50E-08	244207	Y
rs9817910	G	A	0.435399	0.010493	0.00371	3.90E-03	244207	N
rs9836621	C	T	0.480873	0.012987	0.003688	7.00E-04	244207	Y
rs9950528	A	G	0.651077	-0.01193	0.003908	2.00E-03	244207	Y
rs9956387	A	T	0.496773	-0.01762	0.003698	1.90E-06	244207	N
rs9964420	C	A	0.696235	0.032513	0.004029	1.10E-15	244207	Y
rs9997394	G	A	0.708967	0.013667	0.00406	1.00E-03	244207	Y

Supplementary Table 2 - SNP list and female-specific effect estimates of sleep duration SNPs identified in UKBiobank [Dashti et al, 2019]

SNP	effect_allele	other_alle	eaf	beta	se	pval	N
rs915416	C	G	0.289689	0.023004	0.003438	6.10E-11	241350
rs269054	T	A	0.577045	-0.01184	0.003156	1.40E-04	241350
rs61796569	C	T	0.730462	-0.01458	0.003536	1.10E-04	241350
rs12567114	G	A	0.723527	-0.01169	0.003501	9.20E-04	241350
rs2279681*	C	G	0.659907	-0.01544	0.003291	2.20E-06	241350
rs62120041	T	C	0.933589	0.024842	0.006288	1.50E-04	241350
rs374153	C	T	0.157805	0.016296	0.004277	8.40E-05	241350
rs75539574	A	C	0.914038	-0.03739	0.005593	9.50E-11	241350
rs72804080	A	G	0.849894	-0.02174	0.004402	8.00E-07	241350
rs7556815	G	A	0.779458	-0.04256	0.003772	5.50E-29	241350
rs4988235	G	A	0.253166	-0.01509	0.003684	2.70E-05	241350
rs12611523	A	G	0.546115	0.014473	0.003134	1.20E-06	241350
rs4128364	T	C	0.661539	-0.01483	0.003292	1.90E-06	241350
rs4538155	C	T	0.352571	-0.01485	0.003271	1.90E-06	241350
rs11885663	C	T	0.752874	-0.01452	0.003614	5.70E-05	241350
rs10173260	T	C	0.392476	-0.01457	0.00319	8.20E-06	241350
rs112230981	A	G	0.949523	0.030061	0.007172	4.20E-05	241350
rs17732997	C	G	0.569072	0.013842	0.003154	1.30E-05	241350
rs7644809	T	C	0.422402	0.014802	0.00317	8.90E-06	241350
rs13088093	T	G	0.663869	-0.02004	0.003313	1.60E-09	241350
rs7616632	T	G	0.521236	0.017664	0.003128	9.70E-09	241350
rs2192528	A	G	0.480069	0.012955	0.003126	2.20E-05	241350
rs17427571	A	G	0.68445	0.015873	0.003354	1.50E-06	241350
rs35531607	T	C	0.526911	-0.01181	0.003132	1.70E-04	241350
rs13109404	T	G	0.928697	0.036	0.006104	2.10E-09	241350
rs7683893*	T	C	0.503935	-0.01142	0.003162	1.50E-04	241350
rs365663	A	G	0.54667	0.011055	0.003142	2.70E-04	241350
rs460692	C	T	0.137456	0.020151	0.004584	2.80E-05	241350
rs11957190*	T	C	0.368373	-0.01089	0.003228	4.10E-04	241350
rs11135570*	A	G	0.321664	-0.01577	0.003356	6.00E-06	241350
rs56372231	C	T	0.665737	-0.01773	0.003307	5.30E-08	241350

rs180769	T	C	0.424457	0.012827	0.003162	4.10E-05	241350
rs11567976	C	T	0.429651	-0.01215	0.003144	2.10E-04	241350
rs151014368	G	A	0.793547	-0.01784	0.003882	7.60E-06	241350
rs34556183	A	G	0.720184	0.01734	0.003481	8.40E-07	241350
rs80193650	A	G	0.837447	-0.0187	0.004226	2.10E-05	241350
rs113113059	T	C	0.78025	0.023202	0.003777	3.00E-09	241350
rs9382445	T	C	0.623264	0.017023	0.003219	7.40E-08	241350
rs2231265	A	G	0.227509	-0.01337	0.003716	1.90E-04	241350
rs9345234	A	C	0.421598	-0.01287	0.00317	9.60E-05	241350
rs11155606*	T	C	0.52888	0.011728	0.00313	1.30E-04	241350
rs34731055	C	T	0.818494	-0.01738	0.004056	2.80E-05	241350
rs10273733*	T	C	0.284957	-0.01281	0.003458	2.10E-04	241350
rs2079070	C	G	0.264217	0.016036	0.003541	6.10E-06	241350
rs7806045	T	C	0.754564	0.015986	0.00362	4.50E-06	241350
rs330088	T	C	0.452092	-0.01674	0.003136	1.80E-07	241350
rs73219758	G	A	0.70768	0.014388	0.003438	3.70E-05	241350
rs10973207	G	T	0.842614	-0.02111	0.004308	1.80E-06	241350
rs1776776	T	C	0.874067	0.019855	0.004706	2.00E-05	241350
rs12246842	A	G	0.459424	0.01721	0.003133	8.80E-08	241350
rs10761674	C	T	0.477043	0.010918	0.003123	4.20E-04	241350
rs11190970	G	A	0.798644	0.017926	0.00389	1.00E-05	241350
rs12569901*	C	G	0.862921	0.020629	0.00453	6.30E-06	241350
rs7915425	T	C	0.174574	0.019612	0.004116	3.00E-06	241350
rs1517572	A	C	0.419697	-0.01293	0.003162	8.80E-05	241350
rs4592416	A	G	0.53572	-0.01648	0.003125	6.50E-08	241350
rs11602180	C	T	0.836168	0.017012	0.00422	1.40E-04	241350
rs174560	T	C	0.686366	-0.0104	0.003357	1.80E-03	241350
rs12791153	A	T	0.918475	-0.02637	0.005798	2.60E-06	241350
rs1553132	A	G	0.741413	-0.01523	0.003561	2.70E-05	241350
rs1939455	G	T	0.879604	0.021049	0.004914	2.20E-05	241350
rs7115226	C	A	0.926604	-0.02837	0.006007	3.70E-06	241350
rs1263056	A	G	0.519461	0.015902	0.003137	2.20E-07	241350
rs7951019	T	G	0.967792	-0.03194	0.008985	4.30E-04	241350

rs1057703	T	G	0.853581	-0.01984	0.00442	8.40E-06	241350
rs1073160*	G	A	0.48982	0.010957	0.003138	2.80E-04	241350
rs34354917	C	A	0.710963	0.011209	0.003445	9.60E-04	241350
rs11614986	A	G	0.820356	0.016636	0.004059	4.30E-05	241350
rs4767550	A	G	0.585909	-0.01137	0.003184	5.10E-04	241350
rs308604*	G	A	0.164845	0.017167	0.004229	1.30E-04	241350
rs6575005	T	C	0.757596	0.015461	0.003638	1.60E-05	241350
rs10483350	A	G	0.804932	-0.01872	0.003955	1.60E-06	241350
rs61985058	C	T	0.856253	-0.02167	0.004439	3.40E-06	241350
rs55658675	C	T	0.645368	0.015315	0.003265	8.20E-07	241350
rs11621908	C	T	0.916911	0.025692	0.005718	7.50E-06	241350
rs8038326	A	G	0.726755	0.014863	0.003497	6.80E-06	241350
rs3095508	C	A	0.594124	0.014621	0.003176	3.70E-06	241350
rs4780834*	G	C	0.776382	-0.00779	0.003747	2.20E-02	241350
rs11643715	C	G	0.708383	-0.01248	0.003443	6.30E-04	241350
rs9940646	C	G	0.579259	0.01658	0.003157	2.20E-07	241350
rs8050478	G	A	0.500711	0.019315	0.003117	1.90E-10	241350
rs7503199	C	T	0.733905	0.01273	0.003528	9.10E-04	241350
rs205024	C	T	0.615797	-0.01304	0.003205	4.00E-05	241350
rs2139261	C	G	0.251754	-0.02042	0.003987	1.80E-07	241350
rs1991556	G	A	0.773598	0.015319	0.003754	8.00E-05	241350
rs9903973	C	T	0.466491	0.009806	0.00313	2.10E-03	241350
rs8074498*	T	A	0.417181	0.015143	0.003189	6.00E-07	241350
rs12607679	T	C	0.73809	0.021369	0.003569	3.70E-09	241350
rs10421649	T	A	0.443612	-0.01283	0.003159	4.30E-05	241350
rs2072727	T	C	0.436414	0.014854	0.003148	4.40E-06	241350
rs3788337*	G	A	0.647658	0.011694	0.00327	6.40E-04	241350

*variants not reaching genome-wide significance in UKBiobank were identified in GWAS meta-analysis with CHARGE in Dashti et al, 2018

Supplementary Table 3 - SNP list and female-specific effect estimates of insomnia symptoms SNPs identified in UKBiobank [Lane et al, 2019]

SNP	effect_alle	other_alle	eaf	beta	se	pval	N
rs4751*	G	T	0.576387	-0.01072	0.001999	8.30E-08	245767
rs12405761	A	C	0.569803	0.009453	0.001995	2.10E-06	245767
rs2613503	C	A	0.19827	-0.00757	0.002481	2.30E-03	245767
rs11804386	G	A	0.666433	-0.00798	0.002091	1.30E-04	245767
rs11184946*	C	T	0.583123	-0.0076	0.002003	1.50E-04	245767
rs6664467*	G	A	0.863973	0.005474	0.002881	5.70E-02	245767
rs2644128	C	G	0.449308	-0.0093	0.001983	2.70E-06	245767
rs12713372	T	C	0.433714	-0.00545	0.001992	6.20E-03	245767
rs35881094	T	G	0.572651	-0.01056	0.001999	1.30E-07	245767
rs2192338	G	C	0.2199	-0.0088	0.002386	2.20E-04	245767
rs113851554	G	T	0.94258	-0.05024	0.004397	3.20E-30	245767
rs72826719*	G	A	0.951197	-0.02652	0.00463	1.00E-08	245767
rs62158170	A	G	0.783846	0.01101	0.002407	4.80E-06	245767
rs4577309	A	G	0.469167	0.009646	0.001984	1.20E-06	245767
rs4683301*	A	T	0.400444	-0.0075	0.00202	2.10E-04	245767
rs10865954	T	C	0.334332	0.009155	0.002092	1.20E-05	245767
rs4688760	C	T	0.309931	-0.01133	0.002138	1.20E-07	245767
rs55946513	C	T	0.934353	0.015559	0.003987	9.50E-05	245767
rs9845387*	C	A	0.959798	0.018019	0.00503	3.40E-04	245767
rs6785034	G	A	0.578777	-0.00452	0.001999	2.40E-02	245767
rs1841625*	A	G	0.567914	-0.00788	0.001992	7.60E-05	245767
rs11097861	A	G	0.284813	-0.01044	0.002189	1.90E-06	245767
rs1430205*	C	T	0.542055	-0.00739	0.001985	2.00E-04	245767
rs28061*	A	G	0.692084	0.010043	0.002151	3.00E-06	245767
rs1592757*	G	C	0.644472	-0.00955	0.002062	3.60E-06	245767
rs7711696*	G	T	0.695396	-0.01289	0.002143	1.80E-09	245767
rs10947690*	A	G	0.737552	-0.00982	0.002243	1.20E-05	245767
rs6932158*	T	C	0.509034	-0.00844	0.001976	1.90E-05	245767
rs314280*	A	G	0.45255	-0.00803	0.001984	5.20E-05	245767
rs3824081	T	C	0.475619	0.00801	0.00198	5.20E-05	245767
rs302165	G	A	0.216647	0.005405	0.002397	2.40E-02	245767

rs6593005*	A	G	0.25953	-0.00952	0.002253	2.40E-05	245767
rs10280045*	C	G	0.426918	-0.00845	0.002009	2.60E-05	245767
rs17151854*	G	T	0.847077	-0.01293	0.00275	2.60E-06	245767
rs11793831	G	T	0.58258	0.004111	0.002005	4.00E-02	245767
rs11793074	A	G	0.852655	0.004522	0.00278	1.00E-01	245767
rs10156602	A	G	0.6379	0.010785	0.002069	1.90E-07	245767
rs2296580	G	T	0.702195	0.009433	0.002154	1.20E-05	245767
rs11191595	A	C	0.937145	0.026903	0.004274	3.10E-10	245767
rs10838708*	G	A	0.540575	0.007109	0.001998	3.70E-04	245767
rs324017	A	C	0.294459	0.010747	0.002168	7.10E-07	245767
rs2956278*	A	G	0.785694	-0.01306	0.002403	5.50E-08	245767
rs68094047*	C	T	0.749119	-0.01177	0.002281	2.50E-07	245767
rs1923770	T	A	0.382871	0.008312	0.002035	4.40E-05	245767
rs1031654*	C	A	0.200699	0.0142	0.002466	8.50E-09	245767
rs4886140*	A	G	0.333615	-0.01005	0.002113	2.00E-06	245767
rs2147141	C	G	0.456318	-0.00948	0.002006	2.30E-06	245767
rs11635495*	T	C	0.485806	-0.00983	0.001977	6.60E-07	245767
rs4886860	G	C	0.233356	0.011911	0.002333	3.30E-07	245767
rs17139246*	T	C	0.610798	-0.01036	0.002039	3.70E-07	245767
rs1544637*	T	C	0.487796	0.00886	0.00198	7.60E-06	245767
rs3104778*	A	G	0.589325	0.009345	0.002009	3.30E-06	245767
rs2062113*	T	C	0.429187	0.010643	0.001998	1.00E-07	245767
rs17669584	A	G	0.804672	-0.01299	0.002539	3.10E-07	245767
rs11651809	C	G	0.705134	-0.01461	0.002183	2.20E-11	245767
rs1942262	G	A	0.708362	-0.01078	0.00217	6.80E-07	245767
rs11673344*	A	G	0.620064	-0.00838	0.002033	3.70E-05	245767

*variants not reaching genome-significance in relation to any insomnia symptoms in UK Biobank were identified in relation to frequent insomnia in Lane et al, 2018

Supplementary Table 4 - SNP list and female-specific effect estimates of short sleep SNPs identified in UKBiobank [Dashti et al, 2019]

SNP	effect_alle	other_alle	eaf	logOR	se	pval	N
rs7524118	T	C	0.291934	-0.03025	0.008912	0.00067	222223
rs2186122	A	T	0.438719	-0.02317	0.008265	0.005032	222223
rs1256711	G	A	0.723527	0.033971	0.00916	0.000196	222223
rs2820313	A	G	0.658081	-0.0373	0.008425	1.28E-05	222223
rs1380703	A	G	0.617515	-0.03343	0.00896	0.000116	222223
rs7553957	A	C	0.914038	0.042803	0.014804	0.003728	222223
rs2863957	C	A	0.779007	0.05319	0.009833	6.63E-08	222223
rs2014830	C	T	0.69751	0.030047	0.00891	0.000755	222223
rs1700511	G	A	0.735852	-0.02762	0.009518	0.002803	222223
rs1310732	C	T	0.926549	-0.08893	0.01516	5.12E-09	222223
rs1251846	T	C	0.671802	-0.02274	0.00855	0.006913	222223
rs3776864	A	C	0.666586	0.03304	0.008615	0.000128	222223
rs4585442	A	G	0.689101	-0.02664	0.008516	0.002308	222223
rs1421807	T	C	0.990597	-0.157	0.04368	0.000305	222223
rs1266166	C	T	0.737048	-0.03053	0.00949	0.000985	222223
rs9367621	T	A	0.430902	0.024693	0.008027	0.002593	222223
rs9321171	C	T	0.540379	0.031697	0.008175	0.000102	222223
rs1176375	G	A	0.813548	0.034695	0.01046	0.000894	222223
rs1229762	C	T	0.335448	-0.04259	0.008536	6.97E-07	222223
rs6088275	A	T	0.938851	0.070852	0.01704	3.15E-05	222223
rs1607227	G	T	0.704614	0.021224	0.008937	0.01757	222223
rs7939345	T	G	0.207663	0.035367	0.009946	0.000343	222223
rs1738880	A	C	0.89437	-0.05827	0.013658	1.48E-05	222223
rs5977955	T	G	0.553473	0.02501	0.00812	0.002181	222223
rs205024	C	T	0.615797	0.021428	0.008303	0.009868	222223
rs1296346	C	T	0.298539	0.036332	0.008934	6.40E-05	222223
rs5757675	G	T	0.258725	0.039221	0.009407	2.05E-05	222223

Supplementary Table 5 - SNP list and female-specific effect estimates of long sleep SNPs identified in UKBiobank [Dashti et al, 2019]

SNP	effect_allele	other_allele	eaf	logOR	se	pval	N
rs7534398	A	T	0.199991	0.052592	0.01573	0.00088	184999
rs6737318	G	A	0.220835	0.080658	0.014803	7.65E-08	184999
rs5499610	T	C	0.001508	0.536493	0.151372	0.000388	184999
rs1089925	A	G	0.145172	0.057325	0.017646	0.001124	184999
rs7545865	T	C	0.022924	0.18648	0.039147	2.11E-06	184999
rs3751046	G	A	0.146321	0.051643	0.017247	0.003229	184999
rs1781728	G	A	0.480677	-0.03263	0.012594	0.009702	184999
rs1768891	A	T	0.204029	-0.06817	0.016486	3.65E-05	184999

Supplementary Table 6 - SNP list and effect estimates of actigraphy-derived L5 timing SNPs identified in UKBiobank [Jones et al, 2019]

SNP	effect_allele	other_allele	eaf	beta	se	pval	N
rs1144566	C	T	0.97	0.096	0.014	8.00E-12	85,830
rs113851554	T	G	0.057	0.133	0.011	2.00E-35	85,830
rs12991815	C	G	0.424	0.029	0.005	2.00E-09	85,830
rs9369062	A	C	0.708	0.039	0.005	9.00E-14	85,830
rs4882315	T	C	0.507	0.027	0.005	2.00E-08	85,830
rs12927162	G	A	0.277	0.029	0.005	3.00E-08	85,830

Supplementary Table 7 - SNP list and effect estimates of actigraphy nocturnal sleep duration SNPs identified in UKBiobank [Jones et al, 2019]

SNP	effect_allele	other_allele	eaf	beta	se	pval	N
rs2660302	A	T	0.811	0.041	0.006	9.00E-12	85,502
rs113851554	G	T	0.943	0.11	0.011	2.00E-25	85,502
rs62158170	G	A	0.217	0.054	0.006	3.00E-21	85,502
rs17400325	T	C	0.958	0.066	0.012	2.00E-08	85,502
rs72828540	T	C	0.752	0.041	0.005	1.00E-13	85,502
rs9369062	C	A	0.292	0.033	0.005	2.00E-10	85,502
rs2975734	C	G	0.561	0.027	0.005	1.00E-08	85,502
rs13282541	C	T	0.739	0.032	0.005	4.00E-09	85,502
rs2880370	A	T	0.67	0.028	0.005	2.00E-08	85,502
rs800165	T	T	0.343	0.028	0.005	3.00E-08	85,502
rs10138240	G	G	0.514	0.029	0.005	7.00E-10	85,502

Supplementary Table 8 - SNP list and effect estimates of number of nocturnal sleep episodes SNPs identified in UKBiobank [Jones et al, 2019]

SNP	effect_allele	other_allele	eaf	beta	se	pval	N
rs12714404	T	G	0.283	0.037	0.005	1.00E-12	85,502
rs310727	T	C	0.475	0.026	0.005	3.00E-08	85,502
rs55754932	C	A	0.284	0.037	0.005	2.00E-12	85,502
rs9864672	T	C	0.522	0.029	0.005	2.00E-10	85,502
rs4974697	T	A	0.39	0.026	0.005	5.00E-08	85,502
rs7377083	A	C	0.43	0.029	0.005	2.00E-09	85,502
rs749100	A	G	0.582	0.033	0.005	9.00E-12	85,502
rs9341399	C	T	0.936	0.066	0.01	6.00E-12	85,502
rs1889978	C	T	0.85	0.027	0.005	5.00E-09	85,502
rs10233848	A	G	0.478	0.026	0.005	1.00E-08	85,502
rs2141277	G	A	0.293	0.035	0.005	2.00E-11	85,502
rs1124116	A	G	0.73	0.031	0.005	2.00E-09	85,502
rs4755731	G	A	0.431	0.028	0.005	3.00E-09	85,502
rs3751837	C	T	0.781	0.033	0.006	4.00E-09	85,502
rs8045740	G	T	0.868	0.052	0.007	6.00E-14	85,502
rs11078917	A	C	0.279	0.029	0.005	3.00E-08	85,502
rs11082030	T	C	0.725	0.03	0.005	8.00E-09	85,502
rs8098424	G	A	0.6119	0.027	0.005	1.00E-08	85,502
rs76753486	T	C	0.084	0.047	0.008	2.00E-08	85,502
rs429358	T	C	0.848	0.036	0.007	4.00E-08	85,502
rs12479469	A	G	0.342	0.031	0.005	4.00E-10	85,502

Supplementary Table 9 - Statistical test of the proportional hazards assumption

Model	Exposure	Correlation coefficient*	P-value
Multivariable Cox regression - basic model	Chronotype	-0.0083	0.664
Multivariable Cox regression - full adjusted model	Chronotype	-0.0127	0.527
Mendelian randomization Cox regression	Chronotype	0.0037	0.846
Multivariable Cox regression - basic model	Sleep duration	-0.0099	0.604
Multivariable Cox regression - full adjusted model	Sleep duration	-0.0044	0.828
Mendelian randomization Cox regression	Sleep duration	0.0152	0.427
Multivariable Cox regression - basic model	Insomnia	0.0053	0.78
Multivariable Cox regression - full adjusted model	Insomnia	0.0004	0.982
Mendelian randomization Cox regression	Insomnia	0.0022	0.908

*Values represent the Pearson's correlation coefficient (and corresponding P-values) between the first scaled Schoenfeld residual in conventional Cox regression and the rank-normalised natural logarithm of follow-up time.

Supplementary Table 10 - Multivariable and Mendelian randomization Cox regression of the risk of other cancers associated with sleep traits

Sleep trait	Basic model*				Multivariable-adjusted model †				Mendelian randomization analysis**			
	N (cases)	HR	95% CI	P	N (cases)	HR	95% CI	P	N (cases)	HR	95% CI	P
Chronotype (morningness)	156,536 (7,847)	1.00	0.98, 1.02	0.969	143,131 (7,102)	1.00	0.99, 1.02	0.704	156,536 (7,847)	1.05	0.93, 1.17	0.436
Sleep duration	155,933 (7,811)	1.02	1.01, 1.05	0.009	142,818 (7,085)	1.03	1.01, 1.05	0.011	155,933 (7,811)	0.92	0.72, 1.18	0.52
Insomnia	156,870 (7,865)	0.97	0.94, 1.00	0.075	143,383 (7,117)	0.97	0.93, 1.00	0.056	156,870 (7,865)	1.55	0.94, 2.55	0.088

*Adjusted for age, assessment centre and the top 40 genetic PCs

†Adjusted for age, assessment centre, top 40 genetic PCs, degree, body mass index, alcohol intake, smoking, strenuous physical activity, family history, parity, age at menarche, menopause status, use of oral contraceptives and menopausal hormone therapy

**Adjusted for age, assessment centre, top 40 genetic PCs and genotyping chip

Supplementary Table 11 - Multivariable and Mendelian randomization Cox regression of the risk of death associated with sleep traits

Sleep trait	Basic model*				Multivariable-adjusted model †				Mendelian randomization analysis**			
	N (cases)	HR	95% CI	P	N (cases)	HR	95% CI	P	N (cases)	HR	95% CI	P
Chronotype (morningness)	179,737 (3,704)	0.92	0.90, 0.95	1.89x10-10	164,202 (3,164)	0.95	0.93, 0.97	2.16x10-4	179,737 (3,704)	1.15	0.97, 1.35	0.106
Sleep duration	179,053 (3,667)	1.02	0.99, 1.05	0.16	163,844 (3,145)	1.01	0.98,1.04	0.69	179,053 (3,667)	1.01	0.71, 1.44	0.944
Insomnia	180,140 (3,722)	1.11	1.05, 1.16	3.96x10-5	164,504 (3,176)	1.08	1.02,1.14	0.004	180,140 (3,722)	1.10	0.53, 2.26	0.8

*Adjusted for age, assessment centre and the top 40 genetic PCs
†Adjusted for age, assessment centre, top 40 genetic PCs, degree, body mass index, alcohol intake, smoking, strenuous physical activity, family history, parity, age at menarche, menopause status, use of oral contraceptives and menopausal hormone therapy
**Adjusted for age, assessment centre, top 40 genetic PCs and genotyping chip

Supplementary Table 12 - Multivariable and Mendelian randomization logistic regression of the risk of breast cancer associated with sleep traits

Sleep trait	Basic model*				Multivariable-adjusted model †				Mendelian randomization analysis**			
	N (cases)	OR	95% CI	P	N (cases)	OR	95% CI	P	N (cases)	OR	95% CI	P
Chronotype (morningness)	156,454 (7,765)	0.95	0.94, 0.97	1.70x10-7	143,080 (7,051)	0.96	0.94, 0.98	1.49x10-5	156,454 (7,765)	0.87	0.77, 0.97	0.014
Sleep duration	155,857 (7,735)	1.02	1.00, 1.04	0.035	142,766 (7,033)	1.02	1.00, 1.05	0.029	155,857 (7,735)	1.16	0.90, 1.48	0.25
Insomnia	156,785 (7,780)	1.11	1.08, 1.16	1.05x10-10	143,326 (7,060)	1.11	1.07, 1.15	8.41x10-9	156,785 (7,780)	1.09	0.65, 1.84	0.73

*Adjusted for age, assessment centre and the top 40 genetic PCs

†Adjusted for age, assessment centre, top 40 genetic PCs, degree, body mass index, alcohol intake, smoking, strenuous physical activity, family history, parity, age at menarche, menopause status, use of oral contraceptives and menopausal hormone therapy

**Adjusted for age, assessment centre, top 40 genetic PCs and genotyping chip

Supplementary Table 13 - Multivariable and Mendelian randomization Cox regression of breast cancer risk with sleep traits, excluding night shift workers

Sleep trait	Basic model*				Multivariable-adjusted model †				Mendelian randomization analysis**			
	N (cases)	HR	95% CI	P	N (cases)	HR	95% CI	P	N (cases)	HR	95% CI	P
Chronoty	146,542 (2,654)	0.95	0.92, 0.97	2.33x10-4	134,066	0.96	0.93, 0.99	0.006	146,542	0.84	0.69, 1.02	0.083
Sleep	145,993 (2,646)	1.01	0.97, 1.04	0.66	133,782	0.99	0.96, 1.03	0.77	145,993	1.07	0.71, 1.63	0.73
Insomnia	146,856 (2,662)	1.02	0.96, 1.08	0.51	134,300	1.02	0.97, 1.09	0.43	146,856	1.26	0.53, 3.00	0.6

*Adjusted for age, assessment centre and the top 40 genetic PCs
†Adjusted for age, assessment centre, top 40 genetic PCs, degree, body mass index, alcohol intake, smoking, strenuous physical activity, family history, parity, age at menarche, menopause status, use of oral contraceptives and menopausal hormone therapy
**Adjusted for age, assessment centre, top 40 genetic PCs and genotyping chip

Supplementary Table 14 - Mendelian randomization Cox regression analysis of risk of breast cancer with chronotype, using allele score composed of replicated SNPs

Sleep trait	N	Mean number of increasing alleles	SD	Association of allele score with sleep trait*				Mendelian randomization analysis			
				Coefficient (SE)	P-value	R^2	F-statistic	N (cases)	HR	95% CI	P
Chronotyp	156,454	230	9.7	0.017	$<1x10^{100}$	0.0161	2564	151421	0.89	0.71, 1.12	0.31

**Adjusted for age, assessment centre, top 40 genetic PCs and genotyping chip

Supplementary Table 15 - Associations between allele scores and potential confounders in UK Biobank

**Instrument**	**Confounder**	**beta***	**se**	**n**	**p**	
insomniascore	hrt	0.155	0.028	156482	2.99E-08	*
durationscore	age at menarche	0.050	0.010	152421	1.63E-07	*
chronoscore	vig	0.084	0.016	148712	3.61E-07	*
durationscore	bmi	-0.011	0.003	156381	2.28E-04	*
insomniascore	age at menarche	-0.028	0.008	152421	3.98E-04	*
chronoscore	parity	0.089	0.027	156766	1.08E-03	*
insomniascore	degree	-0.076	0.025	155477	2.41E-03	
chronoscore	degree	-0.172	0.060	155477	4.39E-03	
insomniascore	smoking	0.044	0.019	156326	0.022	
insomniascore	bmi	0.005	0.002	156381	0.044	
chronoscore	smoking	-0.090	0.046	156326	0.051	
insomniascore	menopause	0.091	0.047	132177	0.054	
durationscore	menopause	-0.111	0.058	132177	0.055	
durationscore	famhist	0.115	0.062	156848	0.064	
durationscore	alcohol	0.061	0.035	156702	0.076	
durationscore	parity	0.024	0.014	156766	0.081	
insomniascore	nightshift	0.054	0.032	86822	0.091	
durationscore	nightshift	-0.065	0.039	86822	0.095	
chronoscore	age at menarche	-0.030	0.019	152421	0.113	
chronoscore	alcohol	-0.107	0.068	156702	0.114	
durationscore	pill	-0.063	0.041	156578	0.125	
insomniascore	pill	0.046	0.034	156578	0.176	
insomniascore	parity	0.014	0.011	156766	0.231	
insomniascore	famhist	0.059	0.051	156848	0.247	
insomniascore	alcohol	0.032	0.028	156702	0.252	
durationscore	degree	0.026	0.031	155477	0.398	
chronoscore	menopause	0.093	0.113	132177	0.412	
chronoscore	pill	-0.062	0.081	156578	0.445	
durationscore	smoking	0.016	0.024	156326	0.494	
insomniascore	vig	-0.004	0.007	148712	0.532	
chronoscore	hrt	0.041	0.067	156482	0.538	
chronoscore	bmi	-0.003	0.006	156381	0.561	
durationscore	vig	-0.002	0.008	148712	0.791	
chronoscore	famhist	0.025	0.122	156848	0.837	
chronoscore	nightshift	-0.006	0.076	86822	0.937	
durationscore	hrt	0.002	0.034	156482	0.959	

Coefficients are in terms of an average-SNP increase in the allele score per unit/level increase in confounder
*associations surpassing multiple-testing correct p-value threshold of 0.05/36 = 1.39x10-3

Supplementary Table 16 - One-sample MR analysis for sleep traits and breast cancer with and without adjustment for potential confounders

Sleep trait	N	MR effect of sleep trait on risk of breast cancer*			N	MR effect of sleep trait on risk of breast cancer (adjusted for potential confounders**)		
		HR	95% CI	P		HR	95% CI	P
Chronotype (morningness)	151,421 (2,732)	0.85	0.70, 1.03	0.098	136,644 (2,508)	0.87	0.71, 1.06	0.15
Sleep duration	150,845 (2,723)	1.06	0.70, 1.59	0.78	136,330 (2,503)	1.04	0.67, 1.61	0.87
Insomnia	149,005 (2,740)	1.37	0.59, 3.20	0.47	139,402 (2,513)	1.35	0.54, 3.38	0.52

*Adjusted for age, assessment centre, 40 PCs and genotype chip

**Adjusted for age, assessment centre, 40 PCs, genotype chip, hrt, age at menarche, vigorous activity, bmi, parity

Supplementary Table 17 -Two-sample MR for sleep traits and risk of breast cancer

			reast cancer (122,977 cases, 105,974 controls		ER+ Breast cancer (69,501 cases, 105,974 controls)		R- Breast cancer (21,468 cases, 105,974 controls	
Exposure (N)	N SNPs	Method	OR (95% CI)	p-value	OR (95% CI)	p-value	OR (95% CI)	p-value
Chronotype	305*	Inverse-Variance Weighted	0.88 (0.82, 0.93)	6.38E-05	0.86 (0.80, 0.92)	2.32E-05	0.88 (0.80,	0.01
		MR Egger	0.84 (0.71, 1.00)	0.049	0.84 (0.69, 1.01)	0.063	0.74 (0.57,	0.023
		Weighted median	0.92 (0.85, 0.99)	0.028	0.93 (0.86, 1.02)	0.11	0.98 (0.86,	0.78
		Weighted mode	0.96 (0.79, 1.16)	0.64	0.99 (0.79, 1.24)	0.94	1.12 (0.80,	0.51
Sleep duration	82**	Inverse-Variance Weighted	1.19 (1.02, 1.39)	0.03	1.21 (1.01, 1.45)	0.036	1.22 (1.03,	0.025
		MR Egger	1.41 (0.83, 2.40)	0.2	1.64 (0.84, 2.80)	0.166	1.10 (0.60,	0.76
		Weighted median	1.27 (1.07, 1.50)	0.005	1.32 (1.09, 1.60)	0.005	1.17 (0.89,	0.26
		Weighted mode	1.34 (1.05, 1.70)	0.022	1.30 (0.98, 1.73)	0.072	1.26 (0.78,	0.36
Insomnia	50***	Inverse-Variance Weighted	0.80 (0.49, 1.31)	0.37	0.70 (0.40, 1.21)	0.2	0.92 (0.55,	0.76
		MR Egger	0.68 (0.17, 2.72)	0.59	0.62 (0.13, 2.97)	0.56	0.54 (0.13,	0.4
		Weighted median	0.93 (0.49, 1.76)	0.82	0.88 (0.61, 1.29)	0.52	1.07 (0.61,	0.82
		Weighted mode	0.96 (0.70, 1.32)	0.8	0.57 (0.32, 1.02)	0.06	0.93 (0.35,	0.88

*Of the 341 SNPs associated with chronotype, 326 (including 24 SNP proxies) were available in the GWAS of breast cancer (Michailidou et al, 2017).
After harmonization and removal of palindromic SNPs with intermediate allele frequencies, 305 SNPs were included in the MR analysis.
**Of the 91 SNPs associated with sleep duration, 86 (including 5 SNP proxies) were available in the GWAS of breast cancer (Michailidou et al, 2017).
After harmonization and removal of palindromic SNPs with intermediate allele frequencies, 82 SNPs were included in the MR analysis.
*** Of the 57 SNPs associated with sleep duration, 53 (including 4 SNP proxies) were available in the GWAS of breast cancer (Michailidou et al, 2017).
After harmonization and removal of palindromic SNPs with intermediate allele frequencies, 50 SNPs were included in the MR analysis.

Supplementary Table 18 - MR Egger test of directional pleiotropy.

Exposure	Intercept (95% CI)	p-value
Chronotype	0.0008 (-0.0024, 0.0039)	0.87
Sleep duration	-0.0030 (-0.0119, 0.0059)	0.5
Insomnia	0.0017 (-0.0123, 0.0156)	0.82

Supplementary Table 19 - Test of heterogeneity (IVW and MR-Egger).

	IVW		MR-Egger	
**Exposure**	**Q**	**p-value**	**Q**	**p-value**
Chronotype	652	$1.57x10^{-27}$	651	$1.23x10^{-27}$
Sleep duration	223	$2.83x10^{-15}$	222	$2.52x10^{-15}$
Any insomnia	321	$8.62x10^{-42}$	321	$3.86x10^{-42}$

Supplementary Table 20 - RadialMR outlier identification

Sleep trait		IVW outlier				Egger outlier			
	SNP	Q-stat	P	Qsum	Qdiff	Q-stat	P	Qsum	Qdiff
**Chronotype**	rs1421085	84.8855148	3.16E-20	84.8855148	-563.766209	78.7633844	7.00E-19	78.7633844	-556.568488
	rs6718511	40.9672313	1.55E-10	125.852746	-522.798978	38.6947385	4.96E-10	117.458123	-517.873749
	rs11681299	18.8810255	1.39E-05	144.733772	-503.917952	18.2634797	1.92E-05	135.721603	-499.61027
	rs7626335	17.5751438	2.76E-05	162.308915	-486.342809	17.6803098	2.61E-05	153.401912	-481.92996
	rs16939162	16.5983816	4.62E-05	178.907297	-469.744427	16.3115979	5.37E-05	169.71351	-465.618362
	rs7225002	15.2635846	9.35E-05	194.170882	-454.480842	15.8298276	6.93E-05	185.543338	-449.788534
**Sleep duration**	rs1991556	35.5051548	2.54E-09	35.5051548	-186.384073	35.5414577	2.50E-09	35.5414577	-184.010324
	rs12246842	27.032405	2.00E-07	62.5375598	-159.351668	27.108924	1.92E-07	62.6503816	-156.9014
	rs35531607	19.4712338	1.02E-05	82.0087936	-139.880434	18.8754241	1.40E-05	81.5258058	-138.025976
**Insomnia**	rs3104778	214.782902	1.24E-48	214.782902	-105.398577	212.664761	3.60E-48	212.664761	-104.016554
	rs4886860	13.0604923	0.00030159	227.843395	-92.3380851	13.0583519	0.00030194	225.723113	-90.9582026

Supplementary Table 21 - Two-sample MR sensitivity analysis with RadialMR outlier removal

Exposure	Outcome	Method	N SNPs	OR (95% CI)	p-value
Chronotype	Breast cancer	Inverse-Variance Weighted	299	0.90 (0.85, 0.95)	0.0002
		MR Egger		0.92 (0.79, 1.06)	0.24
Sleep duration	Breast cancer	Inverse-Variance Weighted	79	1.22 (1.07, 1.39)	0.003
		MR Egger		1.36 (0.89, 2.10)	0.16
Insomnia	Breast cancer	Inverse-Variance Weighted	48	0.94 (0.72, 1.23)	0.64
		MR Egger		0.62 (0.30, 1.29)	0.2

Supplementary Table 22 -Two-sample MR sensitivity analysis for chronotype and risk of breast cancer to assess potential winner's curse

		Breast cancer (122,977 cases, 105,974 controls)		
Exposure (N)	N SNPs	Method	OR (95% CI)	p-value
Chronotype	221*	Inverse-Variance Weighted	0.85 (0.78, 0.92)	6.38E-05
		MR Egger	0.81 (0.67, 0.98)	0.031
		Weighted median	0.91 (0.84, 0.99)	0.035
		Weighted mode	0.97 (0.80, 1.17)	0.72

*Of the 242 SNPs associated with chronotype, 240 were available in the GWAS of breast cancer (Michailidou et al, 2017).
After harmonization and removal of palindromic SNPs with intermediate allele frequencies, 221 SNPs were included in the MR analysis.

Supplementary Table 23 - Robust adjusted profile score sensitivity analysis (MR-RAPS)

Exposure	Outcome	N SNPs	OR (95% CI)	p-value
Chronotype	Breast cancer	305	0.90 (0.84, 0.96)	8.03x10-4
Sleep duration	Breast cancer	82	1.22 (1.05, 1.42)	0.009
Insomnia	Breast cancer	50	0.85 (0.60, 1.21)	0.374

Supplementary Table 24 -Two-sample MR sensitivity analysis for short and long sleep duration and breast cancer risk

			Breast cancer (122,977 cases, 105,974 controls)	
Exposure (N)	N SNPs	Method	OR (95% CI)	p-value
Short duration (<7 hours)	25*	Inverse-Variance Weighted	0.92 (0.86, 0.99)	0.019
		MR Egger	1.03 (0.83, 1.26)	0.83
		Weighted median	0.90 (0.83, 0.98)	0.015
		Weighted mode	0.90 (0.78, 1.03)	0.15
Long duration (> 8 hours)	7*	Inverse-Variance Weighted	1.24 (0.96, 1.60)	0.10
		MR Egger	0.79 (0.43, 1.47)	0.49
		Weighted median	1.07 (0.96, 1.19)	0.22
		Weighted mode	1.06 (0.96, 1.18)	0.29

*27 SNPs associated with short sleep duration were available in the GWAS of breast cancer (Michailidou et al, 2017).
After harmonization and removal of palindromic SNPs with intermediate allele frequencies, 25 SNPs were included in the MR analysis.
*Of the 8 SNPs associated with long sleep duration, 7 (including 1 SNP proxy) were available in the GWAS of breast cancer (Michailidou et al, 2017).
After harmonization and removal of palindromic SNPs with intermediate allele frequencies, 7 SNPs were included in the MR analysis.

Supplementary Table 25 - Genetic correlations of self-reported and accelerometer measures

		Self-reported measures			Accelerometer measures		
		Chronotype	Any insomnia	Sleep duration	L5 timing	Sleep duration	Sleep fragmentatior
Self-reported measures	Chronotype	1	-0.0143	-0.0478	0.9029	-0.0062	0.0118
	Any Insomnia		1	-0.5186	-0.0129	-0.1157	0.0947
	Sleep duration			1	0.0182	0.4296	0.1138
Accelerometer measures	L5 timing				1	0.0793	0.0108
	Sleep duration					1	-0.2117
	Sleep fragmentation						1

Supplementary Table 26 -Two-sample MR for accelerometry-derived sleep traits and risk of breast cancer

Exposure (N)	N SNPs	Method	Breast cancer (122,977 cases, 105,974 controls) OR (95% CI)	p-value
L5 timing	4*	Inverse-Variance Weighted	1.04 (0.78, 1.38)	0.78
		MR Egger	1.32 (0.80, 2.16)	0.38
		Weighted median	1.10 (0.89, 1.37)	0.36
		Weighted mode	1.20 (0.96, 1.49)	0.21
Nocturnal sleep duration	8**	Inverse-Variance Weighted	1.16 (1.02, 1.32)	0.024
		MR Egger	1.34 (0.94, 1.92)	0.15
		Weighted median	1.16 (0.97, 1.37)	0.1
		Weighted mode	1.14 (0.89, 1.47)	0.32
Number of nocturnal sleep episodes	21***	Inverse-Variance Weighted	1.14 (1.00, 1.30)	0.057
		MR Egger	1.76 (0.96, 3.24)	0.083
		Weighted median	1.07 (0.92, 1.26)	0.37
		Weighted mode	0.88 (0.58, 1.34)	0.55

*Of the 6 SNPs associated with L5 timing, 5 (including 1 SNP proxy) were available in the GWAS of breast cancer (Michailidou et al, 2017).
After harmonization and removal of palindromic SNPs with intermediate allele frequencies, 4 SNPs were included in the MR analysis.
**11 SNPs associated with nocturnal sleep duration were available in the GWAS of breast cancer (including 1 SNP proxy) (Michailidou et al, 2017).
After harmonization and removal of palindromic SNPs with intermediate allele frequencies, 8 SNPs were included in the MR analysis.
*** 21 SNPs associated with number of nocturnal sleep episodes were available in the GWAS of breast cancer (Michailidou et al, 2017).
After harmonization and removal of palindromic SNPs with intermediate allele frequencies, 21 SNPs were included in the MR analysis.

Supplementary Table 27 -Two-sample MR for accelerometry-derived L5 timing and risk of breast cancer using chronotype-derived SNPs as instruments

Exposure (N)	N SNPs	Method	Breast cancer (122,977 cases, 105,974 controls) OR (95% CI)	p-value
L5 timing	305	Inverse-Variance Weighted	0.86 (0.78, 0.95)	0.003
		MR Egger	0.94 (0.80, 1.10)	0.44
		Weighted median	1.01 (0.90, 1.14)	0.83
		Weighted mode	1.07 (0.21, 5.38)	0.93

Supplementary Table 28 - Strengths and limitations of epidemiological approaches applied in this study

	Multivariable Cox regression of incident cases	Multivariable logistic regression of prevalent and incident cases	One-sample Mendelian randomization	Two-sample Mendelian randomization
**Strengths**				
	Potential for reverse causation minimized	Improved sample size and power	Potential for reverse causation minimized	Potential for reverse causation minimized
	Permits thorough evaluation of confounders	Permits thorough evaluation of confounders	Genotypes assumed to be randomly distributed with respect to confounders	Genotypes assumed to be randomly distributed with respect to confounders
	Allows for estimation of incidence (hazard) rate of disease		Allows for estimation of incidence (hazard) rate of disease if implemented in a Cox regression framework	Improved sample size and power
			Permits thorough evaluation of confounders to test above assumption	Flexibility and enhanced power to perform an array of sensitivity analyses e.g. pleiotropy-robust methods
			Allows for investigation of subsets of participants	
**Limitations**				
	Susceptibility to unmeasured or residual confounding	Susceptibility to unmeasured or residual confounding	Low power and therefore imprecise causal estimates	Unable to thoroughly evaluate individual-level confounding factors
	Low power	Possibility of reverse causation	Weak instrument bias (towards observational estimate)	Weak instrument bias (towards null)
	Potential for selection bias due to study sampling differential diagnosis	Potential for selection bias due to study sampling	Winner’s curse where genetic variants identified in same dataset as applied in MR analysis may bias estimates upwards	Winner’s curse where discovery GWAS used to estimate SNP-trait association may overestimate effect of genetic instrument relative to the exposure and bias causal estimate towards the null
	Measurement error in exposure and regression dilution bias	Measurement error in exposure and regression dilution bias	Horizontal pleiotropy	Horizontal pleiotropy
			Potential for selection bias due to study sampling	Investigation of subset of participants in an MR framework requires new GWAS to be performed
